# Precision Nanomedicine with Bio-Inspired Nanosystems: Recent Trends and Challenges in Mesenchymal Stem Cells Membrane-Coated Bioengineered Nanocarriers in Targeted Nanotherapeutics

**DOI:** 10.3390/jox14030047

**Published:** 2024-06-24

**Authors:** Mirza Salman Baig, Anas Ahmad, Rijawan Rajjak Pathan, Rakesh Kumar Mishra

**Affiliations:** 1 Anjuman-I-Islam Kalsekar Technical Campus School of Pharmacy, Sector-16, Near Thana Naka, Khandagao, New Panvel, Navi Mumbai 410206, Maharashtra, India; mirzasalman.pharma@gmail.com; 2 Julia McFarlane Diabetes Research Centre (JMDRC), Department of Microbiology, Immunology and Infectious Diseases, Snyder Institute for Chronic Diseases, Hotchkiss Brain Institute, Cumming School of Medicine, Foothills Medical Centre, University of Calgary, Calgary, AB T2N 4N1, Canada; 3 S.B.S.P.M’s B. Pharmacy College, Ambajoagi, Beed 431517, Maharashtra, India; pathanrijwan4610@gmail.com; 4 School of Health Sciences and Technology, University of Petroleum and Energy Studies (UPES), Bidholi, Dehradun 248007, Uttarakhand, India; rakeshk.mishra@ddn.upes.ac.in

**Keywords:** nanomedicine, drug delivery, cell membrane-based nanocarriers, mesenchymal stem cells, personalized nanomedicine, precision medicine

## Abstract

In the recent past, the formulation and development of nanocarriers has been elaborated into the broader fields and opened various avenues in their preclinical and clinical applications. In particular, the cellular membrane-based nanoformulations have been formulated to surpass and surmount the limitations and restrictions associated with naïve or free forms of therapeutic compounds and circumvent various physicochemical and immunological barriers including but not limited to systemic barriers, microenvironmental roadblocks, and other cellular or subcellular hinderances—which are quite heterogeneous throughout the diseases and patient cohorts. These limitations in drug delivery have been overcome through mesenchymal cells membrane-based precision therapeutics, where these interventions have led to the significant enhancements in therapeutic efficacies. However, the formulation and development of nanocarriers still focuses on optimization of drug delivery paradigms with a one-size-fits-all resolutions. As mesenchymal stem cell membrane-based nanocarriers have been engineered in highly diversified fashions, these are being optimized for delivering the drug payloads in more and better personalized modes, entering the arena of precision as well as personalized nanomedicine. In this Review, we have included some of the advanced nanocarriers which have been designed and been utilized in both the non-personalized as well as precision applicability which can be employed for the improvements in precision nanotherapeutics. In the present report, authors have focused on various other aspects of the advancements in stem cells membrane-based nanoparticle conceptions which can surmount several roadblocks and barriers in drug delivery and nanomedicine. It has been suggested that well-informed designing of these nanocarriers will lead to appreciable improvements in the therapeutic efficacy in therapeutic payload delivery applications. These approaches will also enable the tailored and customized designs of MSC-based nanocarriers for personalized therapeutic applications, and finally amending the patient outcomes.

## 1. Introduction

In organisms that are composed of more than one kind of cell, stem cells have been described as undifferentiated cells in the body which can differentiate into several types of functional cellular lineages [1,2,3]. Stem cells are distinct from other types of cells in the aspect that they are capable of self-renewal through the process of cell division. The ability of stem cells in general and mesenchymal stem cells in particular for the self-regeneration, division and production of differentiated progenies, differentiation, and replication into new types of cells with equivalent potency are other characteristics that define their substantive properties [4,5,6]. Native mesenchymal stem cells, despite being thought of as extremely rare cells in the past, are rather common in vivo. Mesenchymal stem cells have been reported in earlier research studies to have rejuvenating and restorative properties in a variety of diseases [7,8,9], including but not limited to, repairing of cartilages in osteoarthritis [10], remodeling and restoration of the performances of the myocardial-infarcted hearts [11], and chronic atrophic gastritis [12] etc. Furthermore, on the global scale, clinical trials exploring the effectiveness of mesenchymal stem cells in management of the ailments have been presently underway, with a focus on autoimmune disorders [13,14], Crohn’s disease [15], grafts versus host diseases [16,17], multiple sclerosis [18], systemic lupus erythematosus [19,20], and systemic sclerosis [21,22] etc.

For example, in a study where haploidentical mesenchymal stem cells were transplanted in a patient suffering from severe treatment resistant grade IV of acute graft versus host disorder of the gut and liver, a striking clinical outcome was observed and the patient was quite well even after some years and clinical outcomes suggested that mesenchymal stem cells could exert an immunosuppressive effect in vivo well [23]. In other clinical reports, refractory luminal Crohn’s disease and fistulizing Crohn’s disease were treated with autologous bone marrow-derived mesenchymal stem cells and this treatment with mesenchymal stem cells was without side effects, lead to decrement in the clinical disease activity index, resulted into rectal mucosal healing and it was concluded that this therapy by mesenchymal stem cells is a safer and feasible approach for Crohn’s disease therapy [24,25]. The outcome of another clinical study for the therapy of multiple sclerosis indicated that mesenchymal stem cells do not exhibit any of the serious adverse events and improvements in the visual acuity and visual evoked response latency and increment in the optic nerve area were observed in this therapeutic regimen [26]. In other clinical studies, mesenchymal stem cells have been employed for the speedy recovery of hematopoiesis in the patients of advanced cancers when these patients were given high doses of chemotherapies. The outcomes of these clinical studies suggested that these mesenchymal stem cell-based therapies were non-toxic and led to improvements in the neutrophils count. Speedy hemopoietic recovery was seen and the treatment approach seems to be feasible and safe with the positive impact on the blood profiling of the cancer patients [27,28].

Because of these characteristic features, stem cells can be employed in clinical settings for the treatment of several illnesses [29,30]. Nonetheless, most of the data regarding stem cell-based therapeutics for diseases originates from small-scale randomized trials that are seldom successful to produce meaningful treatment benefits. Furthermore, in clinical treatment, stem cell-based therapy may sometimes raise the risks of immunological rejection phenomena as well as malignant transformation [31,32].

Targeted delivery of the drug payload is a method which helps the patients receive systemic administration of the medications and pharmacological compounds into particular tissues or organs while minimizing the concentrations of the medication that accumulates in healthy tissues [33,34]. Extended systemic retention capability, evasion of the immunological barriers, biological barrier-breaking capacities, preservation of the pharmacologically active molecules from degradation, targeted drug payload delivery, and controlled drug release are all desirable characteristic features of an efficient drug delivery platform [35,36]. The incorporation of these characteristic features through methods like PEGylation etc. and the idea of targeted delivery of drugs was born out of stimulus-responsiveness, surface alterations and customizations, the use of nanoscale drug delivery carriers, and cellular- or tissue-based specific targeting entities like ligands, aptamers, or antibodies [37,38].

Globally, mesenchymal stem cells have continuously been employed as therapeutics for the treatment of a wide range of diseases in numerous clinical trials [39,40,41,42]. Due to their strong immunomodulatory as well as anti-inflammatory features, they have been extensively used [43]. Moreover, drug-loaded and diagnostic nanocarriers have been delivered for the particular targeting sites via these mesenchymal stem cells [44]. For example, B16F10 melanoma bearing mice’s lung and sites of the metastatic tumors had incorporated PLGA-DOX-loaded mesenchymal stem cells, which have exhibited enhanced anti-tumor efficacies [45,46]. Scientific researchers whose reports have offered fascinating perspectives on cellular therapeutic paradigms for various disorders have shown a great deal of interest in mesenchymal stem cell-based nanocarriers and nanomedicines due to their promising features, which include the regenerative properties of mesenchymal stem cells supplemented with their ability to differentiate into diverse cell lineages [47,48]. The unequalled capacity of mesenchymal stromal cells for their adherence to the pathological tissues render them promising drug delivery platforms for targeted delivery of therapeutic payload [49,50]. It has been evidenced previously that mesenchymal stem cells possess the capability for absorption and they then subsequently release the chemotherapeutic drugs (e.g., Paclitaxel) as well as causing the inhibition of the growth of subcutaneous tumor xenografts [51,52].

Adult stem cells, more specifically mesenchymal stem cells, also produce nanovesicles, just like other cell types. It was previously considered that the small molecules that mesenchymal stem cells could secrete included chemokines, cytokines, and growth factors [53,54]. On the other hand, mesenchymal stem cells have been reported to release small nano-sized vesicles in reaction to a wide array of chemical signals, mechanical, and environmental stimuli. Nanovesicles derived from mesenchymal stem cells are loaded with markers which have been quite specific to mesenchymal stem cells, including CD105, CD90, CD29, CD73, CD44, and KIT (CD117). Several other cell types in the nearby or distant environment can be influenced and become altered by these vehicles. Primary CD34+ cells and hematopoietic stem cells derived from umbilical cord blood have been demonstrated to proliferate when exposed to nanovesicles derived from mesenchymal stem cells [55,56,57]. They also affect the fate of the hematopoietic system and stop human stem cells from going through apoptosis. Conversely, mesenchymal stem cell-derived nanocarriers suppress the growth of B lymphocytes and exhibit immunosuppressive properties by inhibiting the activity of natural killer cells and the production of interferon gamma [58,59,60]. In conclusion, mesenchymal stem cell-based nanocarriers have a variety of uses and can affect the traits and behavior of other recipient cells and tissues in a range of situations, such as immune modulation, tissue repair, cancer progression, and embryonic development including but not limited to a wide array of diseases and disorders [61,62,63].

Several recent review papers have been published which discuss various aspects of mesenchymal stem cell-based nanocarriers and several advancements in this arena. For example, Mian Wang and coworkers [64] have discussed recent advances in the context of mesenchymal stem cells membrane-coated nanocarriers for biomedical uses, especially in for their anti-cancer and anti-inflammatory applications. They have explained the aspects like mesenchymal stem cell membrane and their receptors, alterations and tailoring of mesenchymal stem cell membrane for homing of various types of nanoparticles, fabrication of these nanomedicine for loading into mesenchymal stem cells etc. However, this review has lacunae in terms of the mechanism of action of these MSC-based nanoformulations along with their metabolism, biotransformation, toxicity and safety concerns, etc. Another study by Wenjing Liu et al. [65] has discussed various advances of various specific cellular membrane types, which have been obtained from various kinds of cells including stem cells, immunological cells, platelet cells, neutrophils, red blood cells, and cancerous cells. They have thoroughly described extraction of the cellular membranes by various techniques and then their applications in anti-cancer therapeutics. However, their perspective lacks in terms of the safety and toxicity of these membrane-based nanocarriers and various other metabolic aspects.

In another recent report by Weiyue Zhang and co-workers [66], they have reviewed stem cells membrane-based targeted drug delivering systems specifically in case of anti-cancer medicines. They have discussed the underlying mechanism for homing these nanosystems inside the cancers, by the optimized modifications of the membranes of the mesenchymal stem cells. They have discussed various aspects including the enhanced drug loading capacities of these nanocarriers, enhanced biocompatibility, selective targeting of tumor tissues and various other aspects. However, their report is lacking in terms of the several aspects of metabolism, biotransformation and excretion of these stem cell membrane based nanocarriers. Furthermore, their report focuses on specific application of these nanocarriers in anti-cancer applications, rather than the broader areas of application of these cellular membrane-based nanocarriers including immunotherapy and regenerative medicine. Likewise, another comprehensive review of mesenchymal stem cell-membrane based drug delivery system by Wu et al. [67] focuses on drug and gene delivery strategies by elucidating several aspects which include employing mesenchymal stem cell-based systems as gene carriers, their targeting abilities, their use as drug payload carrying systems and improving their homing capabilities. They have also focused on various aspects of metabolism, formulation approaches and their applications in bio-imaging and photodynamic therapy. However, their report lacks in the wide range of applications of these mesenchymal stem cell-based nanoformulations in a variety of diseases and disorders. In the present report, the authors have explained in detail several of these aspects which have been lacking or have been touched upon very briefly in the currently available literature.

The numerous biological activities of mesenchymal stem cells are present in nanoparticles coated with their membranes, and the use of carriers is more adaptable. Then, by imitating the mesenchymal stem cells’ capacity for targeting, covering nanoparticles with membranes from mesenchymal stem cells not only improves their biocompatibility but also optimizes their therapeutic efficacy.

## 2. Preparation and Characterization of MSC Membrane-Coated Nanocarriers

### 2.1. Isolation of MSC Membrane

The bilayer of phospholipids that makes up the cell membrane is composed of various glycoproteins, polysaccharides, and integral membrane proteins [68]. The use of a pure cell membrane makes sense since it enhances the development of cell membrane-coated mimics by maximizing the effectiveness and uniformity of surface coatings while imitating as many of the functions of the original cell as possible. Bone marrow, umbilical cord, or adipose tissue can all be used to collect mesenchymal stem cells, which are less invasive and produce higher results [69]. The manufacture of cell membrane-coated nanoparticles mainly involves three processes: nanoparticle cores are manufactured, cell membrane-derived vesicles are created and separated, and then cell membrane-derived vesicles and nanoparticle cores are fused together [70]. To remove the cytoplasm from mesenchymal stem cells, they are first lysed using hypotonic lysis solutions [71], or by repeatedly freezing and thawing them [72]. Subsequently, they may undergo homogenization or sonification in order to reduce their size. Second, to make the mesenchymal stem cell membrane, the product is extracted using centrifugation and then repeatedly extruded from porous polycarbonate membranes with pore widths ranging from 200 to 400 nm [73], thus raising mesenchymal stem cell membranes. Maintaining cell membrane-derived vesicles below −20 °C is necessary to ensure membrane protein stability over long term [74].

The delicate extraction of cell membranes frequently involves the processes of cell lysis and membrane purification, which aid in preventing denaturation of membrane proteins. Removing a cell membrane from different cell types while reducing cytosolic, mitochondrial, and nucleus contamination is the process of isolating a cell membrane. Cell lysis is often performed in small amounts prior to the separating of cell membranes [75]. After homogenizing the cells using sonication to break them up, the mixture’s nucleus and plasma membranes are separated using high-speed gradient centrifugation. The membrane-rich portion is sonicated to create membrane vesicles, which are then passed through a polycarbonate membrane to create nanovesicles, following another wash with isotonic buffers [76]. Many methods, including treating the cells with a hypotonic solution and repeatedly freezing and thawing them, are employed to lyse the cells [72], and/or mechanical rupture (such as extrusion, ultrasound). Discontinuous sucrose gradient centrifugation is used to remove soluble proteins, intracellular biological macromolecules, intracellular vesicles, and the cell nucleus from pure cell membranes. The refined membranes are extruded through polycarbonate porous membranes with nanopores to form nanovesicles [76].

### 2.2. Coating of the Nanoparticle Cores by Membrane Nanovesicles

#### 2.2.1. Extrusion

Extrusion involves continuously sliding membrane vesicles and nanoparticle cores through polycarbonate membranes with varying hole diameters to achieve the desired particle size. Usually, this process is carried out multiple times [77]. In order to thoroughly enclose the nanoparticles, more cell membranes are typically employed than is necessary. Coating polymer-based nanoparticles with a maximum size of 350 nm is a common application of this technique. Extrusion-produced nanoparticles are more effective in encapsulating drugs and have a consistent size distribution. However, this labor-intensive approach is not practical for large-scale industrial applications. Vesicles composed of cell membranes and the cores of nanoparticles are passed through polycarbonate porous membranes throughout the extrusion process, with the pore widths gradually decreasing from 400 nm to 100 nm [70]. The mechanical tension created by the fluidity of the cell membrane during extrusion facilitates the nanoparticles’ penetration through the phospholipid bilayer and their fusion with the membrane vesicles. The diameter of the perforations in the polycarbonate membranes effectively controls the size of the resulting nanoparticles, ensuring a uniform distribution of cell membrane-coated nanoparticles. The biological activity of membrane proteins is greatly preserved by this time-consuming and arduous procedure [78].

#### 2.2.2. Sonication Method

This technique involves fusing together nanoparticle cores and plasma membranes via electrostatic interactions and sonication. Although the simple sonication method can generate core-shell nanoparticles on their own without causing any harm to the cell membrane structure, it is not suitable for the large-scale synthesis of nanoparticles coated with cell membranes. Although sonication and other ultrasonic methods are a good alternative to extrusion [64], they may cause damage to membrane structures. When treated with cell membrane vesicles, ultrasonic waves facilitate the reassembly of the membranes surrounding the nanoparticles. However, optimal parameters such as power, duration, and frequency need to be tuned in order to balance fusion efficiency and reduce protein denaturation. [79].

#### 2.2.3. Microfluidic Electroporation Method

**Figure 1:**
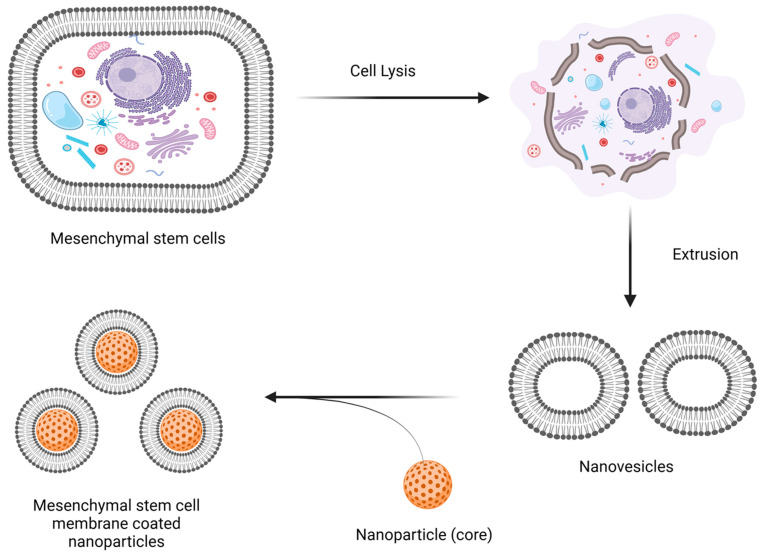
Schematic representation for the formulation steps implicated in the preparation of the mesenchymal stem cell-based nano-therapeutic drug delivery systems.

Cell membranes are perforated via microfluidic electroporation, which uses the electromagnetic energy present in a microfluidic chip [80]. When combining core nanoparticles with cell membrane vesicles, this method becomes very helpful because the created holes make it easier for the vesicles to properly encase the nanoparticles. Throughout this process, parameters like pulse voltage, duration, and flow rate need to be optimized. This is ensued by fully coated, highly reproducible, and uniformly distributed cell membrane-coated nanoparticles. Schematic representation for the formulation steps implicated in the preparation of mesenchymal stem cell-based nano-therapeutic drug delivery systems that ha been depicted in Figure 1.

#### 2.2.4. Flash Nanocomplexation (FNC)

The flash nanocomplexation method [81] involves the preparation of polyelectrolyte solutions containing charged polymers, followed by their simultaneous injection into a mixing chamber of specialized equipment such as a Continuous Impinging Jet Mixer or a Micro vortex Mixer. The high-speed mixing induces rapid and efficient interaction between the oppositely charged polymers, leading to phase separation through polyelectrolyte complexation. This results in the formation of nanoparticles, wherein the polymers and any cargo molecules or drugs become encapsulated within the nanoparticle matrix. Surface modification can be performed to further tailor the properties of the nanoparticles [81]. The resulting nanoparticles are characterized and purified to ensure they meet desired specifications, and the process can be scaled up for industrial-scale production. Overall, FNC provides a rapid, scalable, and environmentally friendly approach to fabricating nanoparticles with precise control over their properties.

### 2.3. Characterization of Cell Membrane-Coated NPs

After the biomimetic membrane-coated nanoparticles are manufactured, appropriate characterization needs to be carried out to ascertain whether the cell membrane coating was effective. Unlike the core nanoparticles, proteins and lipids are the main components of the cell membrane. Transmission electron microscopy is useful to study the core-shell structure of the cell membrane-coated nanoparticles [82]. Simultaneously, the dynamic light scattering method evaluation of the water solubility kinetics of the membrane-modified nanoparticles reveals a minor increase in particle sizes. Furthermore, a successful coating of the cell membrane onto the nanoparticles is shown by the measurement of the zeta potential of the cell membrane-coated nanoparticles matching that of the cell membrane.

### 2.4. Effect of Cell Membrane Coating on Nanoparticle Properties

Coating nanoparticles with cell membranes can significantly impact various properties including size, zetapotential, and polydispersity index (PDI) stability, etc. The size of the nanoparticles can be influenced by the cell membrane coating. Typically, the size of the resulting hybrid nanoparticle will be larger compared to bare nanoparticles due to the addition of the cell membrane layer. The surface charge of nanoparticles can be modified by the cell membrane coating. The charge may become more neutral or negatively charged due to the presence of phospholipids and glycoproteins from the cell membrane.

As reported by Hanze Hu and coworkers [81], increases in particle size after coating were observed through dynamic light scattering, whereas a decrease in particle size and polydispersity index was observed with an increase in the ratio of cell membrane to nanoparticle core (mesoporous silica) when the flash nanocomplexation was used to make the formulation. When stability studies were performed for size, an increase in the particle was observed after two weeks of storage. No major change in morphology was observed after the coating of bare nanoparticles when studied using TEM [81]. Also, an increase in negative-zetapotential was observed with an increase in the ratio of cell membrane to nanoparticle core (mesoporous silica) when the formulation was prepared through flash nanocomplexation and the formulation was studied by the dynamic light scattering method [81]. Similar findings for particle size were also reported by Fang and coworkers [83] where the coating increases the particle size of bare nanoparticles.

The cell membrane coating changed the size of PLGA nano from 225 nm to 247 nm and zeta potential from −55 mV to −43 mV of CMC-NP, when analyzed using DLS technique [84]. An increase in membrane/polymer ratio reduces the particle size [85]. Lang Rao and coworkers [86] found that the lowest core to shell (cell membrane) ratio at which CMC NP demonstrated a steady size was approximately 1 mg UCNPs per 0.2 mL blood. Whereas a negligible difference was observed in the size of CM coated and uncoated nanoparticles, ~25nm. The coating of SiO_2_ NP with CM causes a consistent increase in the hydrodynamic diameter of CMC NP by 10–20 nm and a change in the zeta potential approximately from −37 mV to −32 mV after CM coating [87].

Hui-Wen Chen and coworkers [88] studied cell membrane coating of magnetic nanoparticles. A sharp size distribution was observed before and after membrane cloaking of nanoparticles, indicating the monodisperse nature of the polymeric cores. Although an elevation of zeta potential was observed. The TEM images of the CM-coated NP showed a roughly 20 nm increase in diameter compared with bare NPs [89,90]. The stability of inorganic NPs and their resistance to enzymatic degradation can be improved using membrane coating [90].

Most of the coatings caused an increment of around 10 to 30 nm to the diameter of the nanoparticles [79]. Microfluidic electroporation can produce CMC NP with uniform sizes. Its high reproducibility also guarantees the potential for large scale-up production of CMNPs with enhanced colloidal stability [91,92]. The microfluidic electroporation method exhibits better colloidal stability [93] when compared with the co-extrusion method.

The extrusion and sonication technique is laborious and not economical for use at an industrial scale [94]. When contrasted with alternative approaches, employing microfluidic systems offers clear benefits in minimizing the loss of surface membrane proteins and preserving membrane integrity [95]. The sonication process is suitable for NPs obtained by extrusion and involves less material loss [78]. The flash nano-complexation involves swiftly blending nanoparticle cores with membrane nanovesicles and allowing them to assemble autonomously within a micro-mixing chamber [81].

## 3. Classification of Mesenchymal Stem Cell Membrane-Based Nanocarriers

### 3.1. Lipid Based Nanocarriers

Lipid nanoparticles have been used for targeting different diseases because of their high permeability and high efficiency. Various types of lipid carriers are used for the incorporation of drugs to target the brain, lungs, eye, and tumor cell etc. A novel approach of coating mesenchymal stem cells to the membrane of lipid nanocarriers is used to enhance the efficiency of targeting.

Various research works have been carried out for the development of nanoparticles coated with mesenchymal stem cells for the enhancement of targeting. Clavreul and coworkers [96] formulated Ferrociphenol lipid nanocapsules using the phase inversion temperature approach to target brain tumors specifically and guarantee a wide intratumoral distribution of this delivery vehicle in the orthotopic U87MG glioblastoma model. This study validates the potential benefits of combining stem cell therapy with nanotechnology to enhance the local tissue delivery of anticancer medications in glioblastomas. The researcher found combinations of stem cell therapy and nanotechnology for improving the local tissue distribution of anticancer drugs in glioblastoma due to the intrinsic property of stem cells. They found that the optimum uptake of the drug in glioblastoma was similar in both in vitro and in vivo with similar effects and found inhibition of U87MG cell proliferation. The formulation with mesenchymal stem cells showed potential effects in targeting tumors due to intrinsic properties in the body and also a reduction in side effects to other organs as well as less toxicity due to its biodegradability. Misra, Chopra, and Saikia et al. prepared solid lipid nanocarriers of galantamine hydrobromide by employing the microemulsion technique while using the hot homogenization phenomena to treat Alzheimer’s disease. Adult stem cells, such as mesenchymal stem cells produced from bone marrow, are being extensively investigated as a potential supply of neurons to replace lost or damaged cells in a variety of neurological conditions. When glutamine is administered, it shows a lack of therapeutic effect because of poor brain penetration and bioavailability. The formulation of solid lipid nanoparticles overcame the effect of poor bioavailability and the coating of the drug with mesenchymal stem cells improved the efficiency in the treatment of dementia. An evaluation performed in vitro and in vivo produced results that found the effect of solid lipid nanoparticles coated with mesenchymal stem cells to be a prominent drug delivery for targeting [97].

With multiple formulations having been approved by the US FDA, lipid nanoparticles have been the most therapeutically advanced mesenchymal stem cell-based nonviral gene delivering technologies investigated. They can transfer the nucleic acids in a safer and more effective and efficient manner and can remove a major obstacle to the advancement of genetic therapy. But these and other nanoparticles’ lifetime and colloidal stability in blood circulation must be considered in vivo [64].

Lipid nanoparticle (NP) or volatile polymer systems offer a plethora of interesting therapeutic options for the treatment of brain tumors. These nanoparticles exhibit sustained release characteristics at the site of action along with a notable capability for drug encapsulation. Lipid based nanocarriers can be absorbed efficiently without impairing the ability of MIAMI cells to differentiate or survive. Numerous experiments conducted in our lab have demonstrated the potential of using lipid based nanocarriers to treat glioma tumors [98].

The primary hindrances to the optimal utilization of this promising drug are its low lipophilicity, the need for repeated administration, and the cholinergic side effects associated with GH. We overcame these challenges by encasing GH inside of a novel carrier system known as solid lipid nanoparticles. In rats with cognitive impairment, GH-loaded SLNs were found to be substantially more effective in decreasing inflammatory, metabolic, and behavioral parameters than naive GH [97].

### 3.2. Polymeric Nanoformulations

Polymeric nanoparticles, which can take the shape of nanospheres or nanocapsules, can be created by combining biocompatible, nontoxic, and biodegradable polymers of either synthetic or natural origin. Therapeutic drugs are frequently delivered to a particular target region in a controlled release fashion using nanoparticles as nano-drug conjugates. One of the most fascinating areas of research in recent years is nanodrug delivery. The biophysical and metabolic characteristics of nanoparticles influence not only the bioavailability but also the in-vivo distribution of nano-therapeutic agents. The stability of nanoparticles is affected by several parameters, including their size, shape, type of preparation material, and surface properties. Additionally, the stability of nano-drugs is enhanced by phase transition and additive conjugates, while the shelf life of nano-drug conjugates is enhanced by encapsulation with certain polymer stabilizers. Target delivery of nano-drugs is hampered by issues with release kinetics at the targeted site of action, phagocyte system evasion, and biological barrier crossing [99].

The features of polymeric nanoparticles (NPs), which stem from their small size, have garnered significant attention in recent years. Polymeric nanoparticles (NPs) offer several benefits when used as drug carriers, such as the possibility of controlled release, the capacity to shield biologically active compounds from the environment, and enhanced bioavailability and therapeutic index. Both nanospheres and nanocapsules, which have different morphologies, are included in the word “nanoparticle.” The composition of nanocapsules consists of an oily core that dissolves the medicine and a polymeric shell that regulates the drug’s release profile from the core. The continuous polymeric network that underpins nanospheres allows for the retention of drugs inside or adsorbed onto their surface [100].

In order to surface functionalize synthetic nanomaterials and create biomimetic drug delivery systems for the treatment of cancer, several cell plasma membranes have been used. The biological applications of plasma membranes in functionalizing nanocarriers are facilitated by their natural characteristics and easy isolation from the original cells. Mesenchymal stem cells produced from human umbilical cords have demonstrated a preference for malignant lesions and offer several benefits, including minimal immunogenicity, high proliferative capacity, and ease of acquisition. In order to deliver chemotherapy to specific tumors, we created a poly(lactic-co-glycolic acid) nanoparticle with a layer of plasma membrane from umbilical cord mesenchymal stem cells coating on the surface. The functionalization of mesenchymal stem cells plasma membrane improved Poly(lactic-co-glycolic acid) nanoparticle cellular absorption efficiency, Poly(lactic-co-glycolic acid)-encapsulated doxorubicin tumor cell killing efficacy, and most crucially, the ability of doxorubicin encapsulated in Poly(lactic-co-glycolic acid) to kill tumor cells, and most crucially, the nanoparticles’ ability to target tumors and accumulate there. Consequently, these mesenchymal stem cells’ mimicking nanoformulation produced evident apoptosis within tumor lesions and significantly inhibited tumor development. This study showed the high feasibility of such biomimetic nanoformulations in cancer therapy, as well as the significant potential of umbilical cord mesenchymal stem cells plasma membranes in functionalizing nanocarriers with intrinsic tumor-homing properties for the first time [101].

Wang and coworkers formulated the mesenchymal stem cells loaded with paclitaxel encapsulated poly(d,l-lactide-co-glycolide) nanocarriers for orthotopic glioma therapy in rats. Researchers performed a comparative study of poly(d,l-lactide-co-glycolide) nanocarriers and paclitaxel encapsulated with mesenchymal stem cells and found that mesenchymal stem cells are effective for glioma treatment. The investigated major challenge in brain tumor treatment is the migration of drugs towards the glioma cell of cancer therapy. It has been found that when a drug is encapsulated with mesenchymal stem cells, it reduces the migration and restoration. In the case of plan poly(d,l-lactide-co-glycolide) nanocarriers, it was found that by increasing the rate of excretion of the drug faster, the drug is excreted and therefore there is a reduced restoration effect during the therapy. Nanoparticles coated with mesenchymal stem cells increase the effectiveness of the therapy through restoration and decreasing the rate of excretion. Incorporation of chemotherapeutic drug-loaded nanocarriers into mesenchymal stem cells is a promising strategy for tumor-targeted therapy [102].

Gao and coworkers formulated the doxorubicin hydrochloride Gelatin nanogel for the treatment of Human cervical cancer nanogel prepared by the desolvation process and the coating of nanoparticles with mesenchymal stem cells by the co-extrusion technique. The main challenge for doxorubicin hydrochloride is cardiotoxicity. Following the evaluation of the formulation in vitro on HeLa cells as well as in vivo on Female BALB/c nude mice in a cytotoxicity study, it was found that SCMGs-DOX does not cause a toxic side effect in vivo due to their intrinsic properties and biodegradability [103].

### 3.3. Inorganic

Inorganic nanoparticles are more stable, hydrophilic, nontoxic, and biocompatible than organic compounds. High surface area per unit volume, unique optical and magnetic capabilities, and the ability to be functionalized with various specialized ligands to boost their affinity toward target molecules or cells are some of its other unique qualities. Inorganic nanoparticles not only have a controlled release profile for medications, but they also shield pharmaceuticals from deterioration and can reduce dosages and delivery frequency, which greatly reduces the toxicity of medications—particularly cancer therapy. Innovative materials have led to the evolution of medication delivery methods that have reduced adverse effects and increased treatment efficacy. The main applications of nanotechnology in medicine are in the fields of diagnostic processes, nanodrugs and delivery systems, and biomedical implants. Nanotechnology-enabled medicine delivery is anticipated to present the largest commercial possibility. Thanks to recent developments in nanotechnology, there are now more inorganic nanoparticles accessible besides calcium phosphates that offer effective drug delivery matrices. Nowadays, nanoparticles have incredibly complex chemical properties, and many inorganic nanoparticles have been used as drug carriers. Numerous studies have been conducted on the use of inorganic nanoparticles in cancer detection and treatment, and the field’s applications are expanding. Furthermore, there have been some new developments and applications of calcium phosphate, gold, and iron oxide nanoparticles in tissue engineering and drug delivery [104].

Inorganic nanomaterials, including magnetic nanoparticles, gold nanoparticles, graphene, mesoporous silica nanoparticles, quantum dots, and layered double hydroxides, are among the most actively researched topics in the fields of biochemistry, biotechnology, and biomedicine. Targeted drug delivery, cancer therapies, and bioimaging have shown great promise for inorganic nanomaterials due to their facile manufacturing and modification, intrinsic physicochemical qualities, and excellent biocompatibility [64].

Lixu Xie et al. in 2023 formulated the Manganese Dioxide Nanoparticles Umbilical Cord Mesenchymal Stem Cell Membrane of paclitaxel using the Coextrusion technique for the treatment of lung cancer. The paclitaxel drug can be used for the treatment of lung cancer but the plan drug cannot reach the targeted site; therefore, the inorganic nanoparticles are formulated with manganese dioxide and the coating of nanoparticles with stem cell by incubation with transcriptional transactivator peptide-conjugated 1,2-distearoyl-sn-glycero-3-phosphoethanolamine N-methoxy (polyethylene glycol), whose carbon and hydrogen chains were impromptu incorporated on the cellular membrane by employing the lipid-insertional technique. Results of the study found that the cytotoxic activity of the formulation showed a potent effect and safety data in treated mice showed that the formulation of paclitaxel coated with mesenchymal stem cells has no systemic toxicity. Nanoparticles coated with mesenchymal stem cells provide safety due to the intrinsic nature and biodegradability of the mesenchymal stem cells [105].

Li et al. formulated Silica nano rattle doxorubicin anchored with mesenchymal stem cells for targeting tumor cells. Researchers worked to overcome the change which is the low efficiency of nanoparticle drug delivery to targeted sites. It has been found that the intracellular retention time of the silica nanorattle was no less than 48 h, which is sufficient for cell-directed tumor-tropic delivery. In vivo experiments proved that the burdened mesenchymal stem cells can track down the U251 glioma tumor cells more efficiently and deliver doxorubicin with wider distribution and longer retention lifetime in tumor tissues compared with free doxorubicin and silica nano rattle-encapsulated doxorubicin [106]. Some selected examples of these nano-systems and drug delivery agents based on or associated with mesenchymal stem cells have been presented in Table 1.

## 4. Mesenchymal Stem Cell-Based Nanocarriers for Therapeutics and Regenerative Medicines

### 4.1. MSC-Based Nanocarriers in Regenerative Medicine

**Figure 2:**
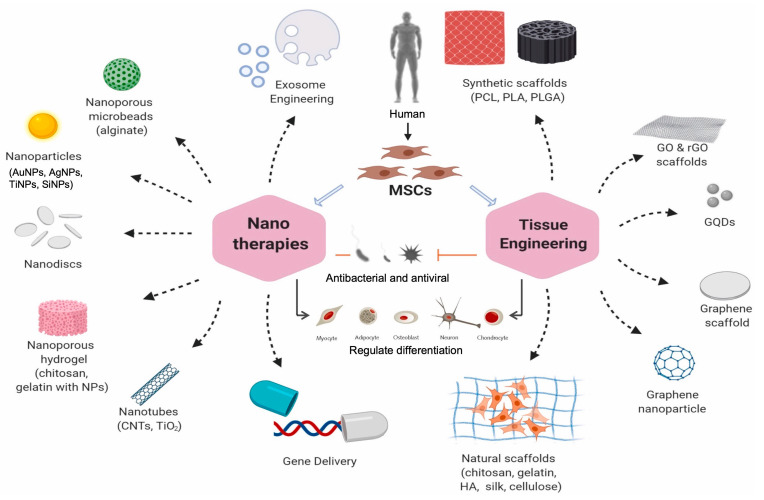
Mesenchymal stem cells have an array of several applications because of their immunomodulatory characteristics and differentiation capabilities; hence, these mesenchymal stem cells have been employed in most stem cell-based research in both preclinical and clinical settings. Reproduced with permission from [108].

A broad biomedical use of mesenchymal stem cell-based nanocarriers is tissue regeneration by nanotherapeutics. These nanocarriers made of mesenchymal stem cells have a dimetric scale of 1 to 100 nm, making them extremely small particles. These mesenchymal stem cell-based nanocarriers find a range of uses due to their tunable optical, electrical, magnetic, and mechanical capabilities, which may be adjusted by modifying particular parameters [108]. For instance, bone marrow-derived mesenchymal stem cells can be driven toward the cardiac lineage by internalizing gold nanoparticles, and cellular adhesion is improved when gold nanoparticles hybridize with the silica nanoparticles and the arginine glycine aspartic acid motifs [109]. Similarly, mesenchymal stem cells differentiate into the neuronal lineage when exposed to dexamethasone-iron oxide nanoparticles, and bone marrow stem cells are supported as an appropriate delivery model for diabetic patients when silica nanoparticles are conjugated with insulin [110]. Furthermore, regeneration of the central nervous system, anti-inflammation at the site of injuries, inhibition of tumors when these mesenchymal stem cell-based nanocarriers become internalized by the corresponding tumor tissue, and cardiac and skeletal disorders are the targets of mesenchymal stem cell-based therapies [111]. A number of mesenchymal stem cell-based therapeutic and regenerative applications have been reported because of their immunomodulatory characteristics and differentiation capabilities; hence, these mesenchymal stems cell-based nano-systems have been used in several pre-clinical and clinical settings as shown in Figure 2.

Immune system, musculoskeletal, neurological, and cardiovascular disorders are treated with mesenchymal stem cell-derived exosomes. For example, an experimental in vitro study established mesenchymal stem cell-derived exosomes as a viable treatment option for osteoarthritis and other cartilage injuries [112,113]. These mesenchymal stem cell-derived exosomes also form collagen II rich, hyaline-like cartilage, which has a regenerative effect on osteochondral defects. Exosome-borne biomolecules have also been shown to be chondroprotective and anti-inflammatory in both in vitro and in vivo studies. Recent studies have demonstrated the potential therapeutic use of mesenchymal stem cell-derived exosomes in models of acute liver injury and liver fibrosis. Specifically, exosomes containing miR-125b can reduce liver fibrosis by suppressing the activation of hedgehog signaling. A novel therapy for osteoarthritis called allogeneic human mesenchymal stromal/stem cells has entered clinical trials. A growing body of research indicates that paracrine signaling is necessary for mesenchymal stem cells therapeutic efficacy. The researchers have looked into how human bone marrow-derived secreted extracellular vesicles aid in the repair of human osteoarthritic cartilage.

Researchers studied the pro-inflammatory genetic expressional alterations by RT-PCR after the mesenchymal stem cell-based nanocarriers treatment of tumor necrosis factor alpha-stimulated osteoarthritic chondrocyte monolayer cultures in order to assess the impact of mesenchymal stem cell-based nanovesicles on osteoarthritic cartilage inflammation. In order to evaluate the effect of mesenchymal stem cell-based nanovesicles on cartilage regeneration, the regeneration cultures of human osteoarthritic chondrocytes were supplemented with mesenchymal stem cell-based nanovesicles. Later, the glycosaminoglycan content of these cultures was measured using the 1,9-dimethylmethylene blue assay. Additionally, type II collagen and proteoglycans (safranin-O) were stained in paraffin sections of the regenerate tissue. They demonstrated that mesenchymal stem cellsbased nanovesicles prevent inflammatory mediators from negatively affecting cartilage homeostasis. Mesenchymal stem cell-based nanovesicles inhibited tumor necrosis factor-alpha-induced collagenase activity and negated tumor necrosis factor alpha-mediated upregulation of cyclooxygenase-2 and pro-inflammatory interleukins when co-cultured with osteoarthritic chondrocytes. In vitro, mesenchymal stem cell-based nanovesicles additionally aided in cartilage regeneration. When mesenchymal stem cells -based nanovesicles were added to chondrocyte cultures obtained from patients with osteoarthritis, these cells produced more type II collagen and proteoglycans. According to the final data, these nanovesicles have a lot of potential as a novel treatment for osteoarthritis and cartilage regeneration. They can also be significant mediators of cartilage repair [114].

Additionally, the mesenchymal stem cell-based exosomes facilitate intercellular communication through the transfer of micro RNAs, which supports recipient neuron axonal growth and retinal ganglion cell survival [115]. Recent research shows that by activating the PI3K/protein kinase B/mechanistic target of rapamycin/glycogen synthase kinase 3β signaling pathway, exosomes designed to be rich in miR-17-92 improve neurological rehabilitation. Similarly, neurite outgrowth and neural plasticity are promoted by mesenchymal stem cells enriched with miR-133b exosomes, indicating a potential therapeutic approach for peripheral nerve damage [116]. Exosomes produced from human mesenchymal stem cells have improved locomotor performance and induced anti-inflammatory action in a rat spinal cord injury model, demonstrating a reparative effect. Furthermore, exosomes produced from human mesenchymal stem cells respond favorably in the cardiac infarct model by enhancing cell proliferation, encouraging neovascularization in vitro, and decreasing the infarct size as determined by monitoring systolic and diastolic blood pressure [117]. According to recent studies, mesenchymal stem cell-derived nanocarriers may be able to treat COVID-19. This can be achieved by either utilizing these nanocarriers unaltered or by adding particular micro RNAs and employing them as drug delivery vehicles. In order to determine whether mesenchymal stem cell-derived nanocarriers are effective in reducing COVID-19 symptoms, numerous clinical trials are being carried out in this area. Mesenchymal stem cell-derived nanocarriers, for instance, may be given to patients by aerosol inhalation; mesenchymal stem cell-based exosomes derived may be employed for the treatment of lung injuries in COVID-19 patients; and exosomes derived from huma bone marrow stem cells may be given intravenously to patients with COVID-19-induced acute respiratory distress syndrome. Putting particular micro RNAs inside these mesenchymal stem cell-based nanocarriers to block SARS-CoV-2’s transcriptional machinery functions as a cell-free therapeutic agent [118].

**Figure 3:**
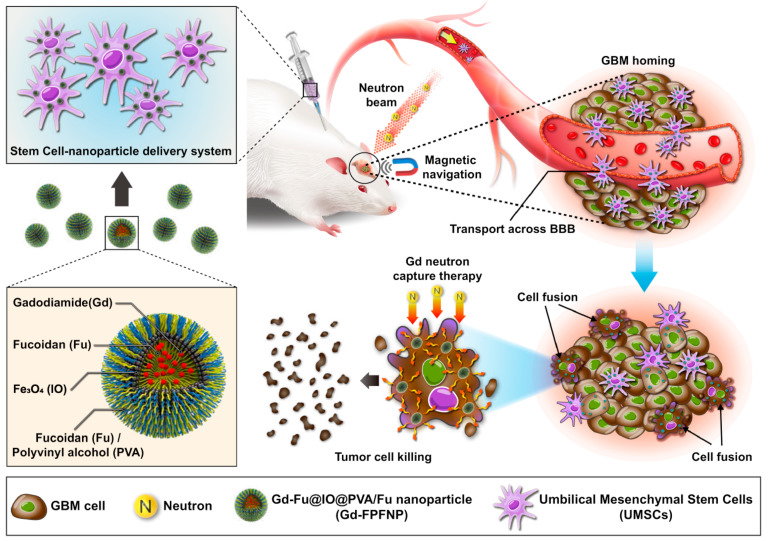
Gadolinium-iron-oxide and polyvinyl alcohol-based nanoparticulate system associated with the mesenchymal stem cells derived from the umbilical cord which possess the capability for crossing the blood brain barrier and can become fused with cancerous cells under magnetic navigation for improved neuron capturing therapeutics (reprinted from [61]).

Because of their capacity for both immune-modulation as well as their regeneration capabilities, extracellular nano-vehicles inferred from mesenchymal stem cells hold great promises as nanotherapeutic platforms for liver diseases and disorders [119,120]. Extracellular nano-vesicles derived from mesenchymal stromal cells have been among the powerful substitutes for whole-cell therapies and are carving their ways into the clinical arena of liver diseases and disorders as nano-therapeutics. In both the clinical samples as well as the animal models, the formation of neutrophil extracellular traps in hepatic tissues has been confirmed as one of the crucial factors for liver ischemia–reperfusion injury. Mesenchymal stem cell-based nanovesicles derived from human umbilical cords may serve to decrease the formation of neutrophil extracellular traps and subsequently enhance liver ischemia–reperfusion injury [121,122]. Researchers have demonstrated mechanistically that functional mitochondria from human umbilical cord-derived mesenchymal stem cell-based nanovesicles are transferred to intrahepatic neutrophils. In order to prevent the formation of neutrophil extracellular traps, this effect leads to the initiation of the mitochondrial fusion, which then restores the mitochondrial status and functions in neutrophils. All of their data point to the therapeutic potential of human umbilical cord-derived mesenchymal stem cell-based nanovesicles for liver ischemia–reperfusion injury by indicating that mesenchymal stem cell-based nanovesicles inhibit the formation of local neutrophil extracellular traps by the transfer of functional mitochondria to the intra-hepatic neutrophils as well as mending the mitochondrial functionalities [123]. The mechanism of action of another stem cell–based nanocarrier system has been depicted in Figure 3 where gadolinium and iron-oxide based nanoparticles have been associated with the mesenchymal stem cells derived from the umbilical cords [61].

(UMSCs) as a bio-NCT agent can cross the blood brain barrier (BBB) and fuse with tumor cells under magnetic navigation for enhanced neuron capture therapy.

Researchers have also investigated several concentrations of gold nanoparticles for assessing the biocompatibility and efficacy in Wharton’s jelly mesenchymal stem cell model and when these nanoparticles were combined with collagen and fluorescein isothiocyanate and characterized by DLS, UV and FTIR Wharton’s jelly mesenchymal stem cells had the better viabilities, higher expression of the receptors, greater distances of the migrations, and lower expression of the apoptosis related proteins. The intracellular uptake of the nanoparticles and mechanism of intracellular uptake further exhibited that these nanoparticles demonstrated cellular uptake through clathrin-mediated endocytosis with improved stabilities in the cells for avoiding the lysosomal degradations and better uptake efficiencies. These also showed better retention capacities and improved tissue integrities in the animal models [124].

Mesenchymal stem cell-derived nanoformulation also showed improved cartilage regeneration in a degenerative relentless osteoarthritis model when researchers employed these for in vitro and in vivo studies. The mesenchymal stem cell-based nanoformulation ameliorated the inflammation and cartilage degeneration and brought down the cartilage loss and bone changes in osteoarthritic conditions. The nanoparticles were formulated based on a cytoplasmic membrane-based nanoformulation approach and contained the mesenchymal stem cell surface characters while lacking the cellular machineries. It imparted the nanocarriers to evade the immunological barriers and to prevent being susceptible to the host-induced alterations in their characteristic features [125].

### 4.2. MSC-Based Nanocarriers in Anti-Cancer Medicine

Targeting tumor cells and/or tumor-associated micro capillaries with the least amount of systemic harm is the aim of cancer treatment. Mesenchymal stromal cells exhibit a unique capacity to adhere to pathological tissues, making them promising agents for targeted medication administration [126,127]. It was demonstrated by researchers that mesenchymal stem cells function as carriers and are capable of absorbing and releasing the chemotherapy drug paclitaxel as well as inhibiting the growth of subcutaneous glioblastoma multiforme xenografts. In order to determine if paclitaxel-loaded mesenchymal stem cells maintain a tropism towards the tumor cells in the brain setting and to define the cytotoxic damage generated by mesenchymal stem cells-driven paclitaxel release in the tumor microenvironment, the researchers employed an orthotopic Glioblastoma model. The mCherry protein was used to fluorescently mark U87MG glioblastoma cells, which were then grafted onto the brains of immunosuppressed rats. The researchers injected green fluorescent protein-expressing mouse mesenchymal stem cells—either loaded or unloaded with paclitaxel—into nearby brain areas. Confocal microscopy was used to evaluate the xenografted brain for paclitaxel-induced cell damage after one week of survival. Overall, mesenchymal stem cell-based carriers showed remarkable tumor tropism. Rats implanted with paclitaxel-mesenchymal stem cells as carriers showed nuclear fragmentation, multi-spindle mitoses, and changes in centrosome number in the nucleus of U87MG cells. These alterations are typical of paclitaxel. The frequency of multinucleated cells formed by numerous spindle mitoses was much greater in the carriers than in the controls when paclitaxel and mesenchymal stem cells were co-grafted into tumors. There were no nuclear changes in the nearby astrocytes or neurons around the tumor [128].

One of the main drawbacks of cancer therapy based on nanoparticulate drug delivery systems is low targeting efficiency. In a paper, mesenchymal stem cells were used as the targeting vehicle and a silica nanorattle as the drug carrier to create an effective method for tumor-targeted medication administration. Without the need for cell preconditioning, a doxorubicin drug delivery system based on silica nanorattle was effectively anchored to mesenchymal stem cells through particular antibody-antigen recognitions at the cytomembrane interface. Each mesenchymal stem cell had up to 1500 nanoparticles put onto it, giving the cells excellent cell survival and tumor-tropic potential. For cell-directed tumor-tropic administration, the silica nanorattle’s intracellular retention duration of at least 48 h is enough. Compared to free DOX and silica nanorattle-encapsulated DOX, in vivo tests demonstrated that burdened mesenchymal stem cells are more effective in locating U251 glioma tumor cells and delivering doxorubicin with a larger dispersion and longer retention lifetime in tumor tissues. The considerable enhancement of tumor-cell apoptosis was further aided by the increased and prolonged intratumoral distribution of DOX. This approach could lead to the development of a strong, broadly applicable targeted tumor treatment approach with low systemic toxicity and great efficacy [106].

**Figure 4:**
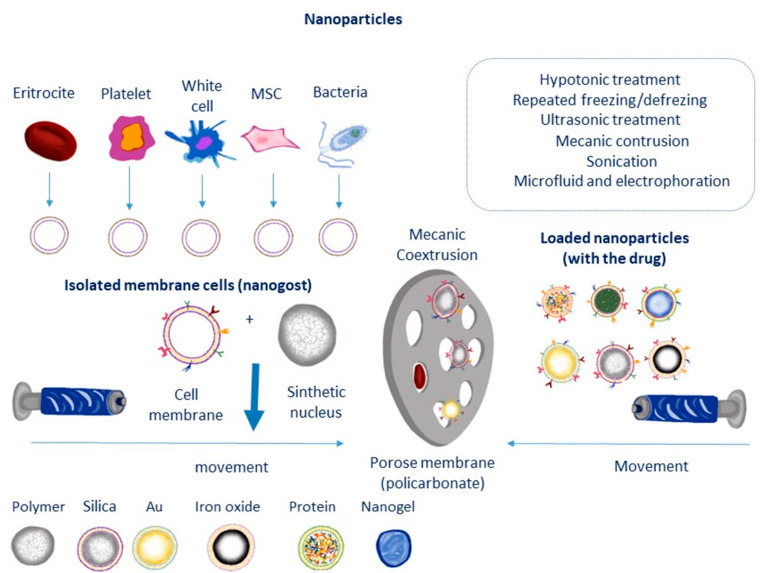
Technologies and physical methods employed in introducing several types of nanoformulations and drug delivery carriers in different types of mesenchymal and other stem cells for anti-cancer therapeutics reprinted from [130].

Gold nanoparticles have been extensively studied for use in photothermal cancer treatment because they can generate heat when subjected to near-infrared light. To improve their tumor-targeting effectiveness and maximize the photothermal impact by adjusting the nanoparticle size, further work has to be done. It has been demonstrated that mesenchymal stem cells can target tumors, assemble pH-sensitive gold nanoparticles in slightly acidic endosomes, and be used in photothermal treatment. Comparing these aggregated structures to pH-insensitive control gold nanoparticles, there was a greater cellular retention, which is crucial for the cell-based administration method. When mesenchymal stem cells loaded with pH-sensitive gold nanoparticles are injected intravenously into tumor-bearing mice, the tumor-targeting efficiency increases 37-fold (5.6% of the injected dose), and the heat generation increases 8.3 °C in comparison to injections of control gold nanoparticles after irradiation. This leads to a markedly improved anti-cancer effect [129]. An example of another study where nanoparticles and mesenchymal stem cell-based therapy has been employed for cancer treatment has been depicted in Figure 4.

The primary pathogenic feature of type 2 diabetes is insulin resistance, which is frequently developed in the elderly. But it is still unknown what fundamental mechanisms underlie insulin resistance associated with aging. Studies have demonstrated that adipocytes, myocytes, and hepatocytes may absorb nanosized exosomes generated by aged mice’s bone marrow mesenchymal stem cells, leading to insulin resistance in both vivo and in vitro. Researchers discovered that the quantity of miR-29b-3p was significantly elevated in the exosomes secreted by aged mice’s bone marrow mesenchymal stem cells using microRNA array tests. Mechanistically, exosomal miR-29b-3p’s downstream target for controlling insulin resistance has been found to be SIRT1 (sirtuin 1). Interestingly, the insulin resistance of elderly mice was markedly improved by using an aptamer-mediated nanocomplex delivery method that down-regulated the expression of miR-29b-3p in bone marrow mesenchymal stem cell-derived exosomes. In the meantime, young mice developed insulin resistance due to bone marrow mesenchymal stem cell-specific upregulation of miR-29b-3p. All of these results pointed to the possibility that exosomal miR-29b-3p produced from bone marrow mesenchymal stem cells could regulate age-related insulin resistance, making it a viable target for therapy [131].

A promising method for magnetic targeting and in vivo tracking of transplanted stem cells is labelling them with magnetic nanoparticles. This is important for enhancing the therapeutic efficacy of cell therapy. Nevertheless, the use of these cutting-edge improvements in stem-cell-mediated regenerative therapy has been hampered by traditional endocytic labelling, which has a brief labelling lifespan and relatively low labelling efficiency. A state-of-the-art magnetothermal technique has been reported by researchers to effectively label mesenchymal stem cells for magnetic resonance imaging tracking and targeted stroke therapy. The technique uses biocompatible γ-phase, ferrimagnetic vortex-domain iron oxide nanorings with superior magnetoresponsive properties as a tracer. This method allows for the safe and effective labelling of γ-phase, ferrimagnetic vortex-domain iron oxide nanorings with up to 150 pg of Fe per cell, without interfering with the proliferation and differentiation of mesenchymal stem cells. This is 3.44 times higher than labelling by endocytosis. In addition to allowing for the long-term tracking of transplanted mesenchymal stem cells over a period of 10 weeks and the ultrasensitive magnetic resonance imaging detection of sub-10 cells, such a high labelling effectiveness also gives transplanted mesenchymal stem cells the capacity to manipulate magnetic fields in vivo. The labelled mesenchymal stem cells enabled magnetic targeting and monitoring for effective replacement therapy with a much lower dosage of 5 × 10^4^ transplanted cells, according to a proof-of-concept study conducted on a rat stroke model. The results of this study have shown how effective the magnetothermal approach can be as a labelling method in the future for use in clinical settings [132].

Without using ionizing radiation, acoustic imaging is accessible and reasonably priced. When applied at high frequencies with excellent temporal resolution, photoacoustic imaging can provide good spatial resolution and contrast compared to a standard ultrasound. Emerging as a photoacoustic contrast agent, Prussian blue nanoparticles have significant optical absorption in the near-infrared spectrum. The researchers created an easy-to-use technique for labelling human mesenchymal stem cells with Prussian blue nanoparticles and using photoacoustic imaging to image the cells. Initially, ferric chloride and K_4_[Fe(CN)_6_] were reacted in the presence of citric acid to create Prussian blue nanoparticles, which were then complexed with the cationic transfection agent poly-l-lysine. With a maximum absorption peak at 715 nm, the poly-l-lysine-coated Prussian blue nanoparticles (nano-complexes) could effectively mark human mesenchymal stem cells. The study employed bright field, fluorescence, and transmission electron microscopy to investigate the cellular uptake of these nanocomplexes. The labelled stem cells expressed CD73, CD90, and CD105 on their surface after effectively differentiating into two downstream lineages of adipocytes and osteocytes. Prussian blue nanoparticle labelling did not affect the viability or proliferation of the labelled cells, and the secretome cytokine analysis showed that the expression levels of 12 distinct proteins were not dysregulated. Following labelling, the optical characteristics of PBNPs were maintained, making them appropriate for the precise and quantitative identification of implanted cells. When scanned at 730 nm, labelled human mesenchymal stem cells showed substantial photoacoustic contrast both in vitro and in vivo; in vivo, the detection limit was 200 cells/μL. As a function of cell concentration, the photoacoustic signal increased, suggesting that the quantity of labelled cells may be measured both before and after cell transplants. This method provides image-guided, real-time brain intraparenchymal injections even through an undamaged skull in hybrid ultrasound/photoacoustic imaging. The 14-day monitoring and identification of 5 × 10^4^ mesenchymal stem cells in living mice was made possible by this labelling and imaging technology [133].

Targeting aging chondrocytes could be a promising therapeutic approach since chondrocytes derived from osteoarthritic cartilage frequently display senescent and aging features. In a recent report, it was suggested that osteoarthritis might be treated by using exosomes made from mesenchymal stem cells obtained from the umbilical cord, together with a regulated release mechanism and the ability to target chondrocytes. This would rejuvenate aging chondrocytes. Extensive functional miRNAs in mesenchymal stem cells from umbilical cords were studied, and the p53 signaling pathway was shown to be the critical component. Exosomes were generated on membranes using a chondrocyte-targeting polymer that was specifically designed for this purpose. The exosomes were then encased within thiolated hyaluronic acid microgels to form a “two-phase” releasing system in a rat model of osteoarthritis cartilage regeneration. The purpose of this was to extend the duration of retention and improve the therapeutic efficacy of mesenchymal stem cells derived from umbilical cords in vivo. In conclusion, this work showed promise for developing a future cell-free osteoarthritis treatment by combining sustained-release and chondrocyte-targeting techniques. It also highlighted the rejuvenating effects of umbilical cord-derived mesenchymal stem cells on osteoarthritis chondrocytes [134].

Because mesenchymal stem cell-based nanomedicines can develop into a wide variety of tissue species depending on the substrate they grow on, they hold great promise for use in the field of regenerative medicine. The capacity of a thin layer of pegylated multiwalled carbon nanotubes spray dried over hot coverslips to affect the proliferation, shape, and ultimate differentiation of human mesenchymal stem cells into osteoblasts was examined by researchers. Their results showed that the uniform layer of functionalized nanotubes promoted cell differentiation more than carboxylated nanotubes or uncoated coverslips by providing a more conducive microenvironment for mesenchymal stem cells. It also did not exhibit any cytotoxicity. It is interesting to note that numerous independent criteria at the transcriptional, protein expression, and functional levels show that cell mesenchymal stem cells differentiation happened even in the absence of additional biochemical inducing factors. When considered collectively, these results indicate that functionalized carbon nanotubes may serve as an appropriate scaffold for a highly selective differentiation into bone [135].

Layek and coworkers have recently explained that anticancer medication non-specific toxicity may be reduced, and therapeutic effectiveness may be enhanced by tumor-targeted drug delivery. Nevertheless, the drug delivery strategies used today rely on the drug carrier’s ineffective passive accumulation inside the cancers. Their approach to tumor targeting is based on the engineering of mesenchymal stem cells with drug-loaded nanoparticles. Our research employing the A549 orthotopic lung tumor model demonstrates that mesenchymal stem cells that have been nanoengineered to carry the anticancer medication paclitaxel settle into tumors and form cellular drug depots that release the drug payload over a few days. Nano-engineered mesenchymal stem cells led to a considerable suppression of tumor development and improved survival even at much lower dosages of paclitaxel. The antitumor effect of mesenchymal stem cells modified by nanotechnology was validated in immunocompetent C57BL/6 albino female mice with orthotopic Lewis Lung Carcinoma tumors. Moreover, leukopenia was induced by paclitaxel solution and paclitaxel nanoparticle treatments, while nano-engineered mesenchymal stem cells had no influence on white blood cell count at dosages that produced equal therapeutic effectiveness. Additionally, compared to the paclitaxel solution and nanoparticle groups, the lung to liver and lung to spleen ratios of paclitaxel for the nano-engineered mesenchymal stem cell group were several times higher, indicating a markedly reduced off-target deposition. In conclusion, our findings show that tumor-specific drug delivery may be effectively facilitated by nano-engineered mesenchymal stem cells, which also markedly increased the anti-cancer efficacy of traditional chemotherapeutic medications [136].

In another study, the possibility of using mesenchymal stem cell-derived exosomes as drug delivery vehicles was assessed. An alternate vesicle for drug delivery might be the synthetically customized exosome mimetics. Exosome mimetic isolation from human mesenchymal stem cells was the goal of researchers. Paclitaxel was added to cells, and exosomal mimetics laden with the drug were separated and tested for their ability to prevent breast cancer. Mesenchymal stem cells generated from human bone marrow were used to isolate exosome mimetics. Mesenchymal stem cells were serially extruded through polycarbonate membrane filters using a mini-extruder, either in the presence or absence of paclitaxel at varying doses in phosphate-buffered saline. After centrifuging mesenchymal stem cells to eliminate debris and filtering the supernatant, exosome mimetics and drug-loaded exosome mimetics were separated by ultracentrifugation. Exosome mimetics without the encapsulated drug payload as well as those containing the paclitaxel were assessed by several techniques like nanoparticle tracking analysis, western blotting, and transmission electron microscopy. Anticancer effects of mesenchymal stem cells derived exosomal mimetics and paclitaxel loaded mesenchymal stem cells derived exosomal mimetics were evaluated with breast cancer cell lines both in vitro and in vivo using the optical imaging system. Exosomal mimetics were isolated by the extrusion method and ultracentrifugation. The membrane markers of the separated vesicles were positive, while the markers of the endoplasmic reticulum and Golgi bodies were negative. Exosomal mimetics produced from mesenchymal stem cells were around 150 nm in size, as determined by nanoparticle tracking analysis, and their shape was validated by transmission electron microscopy. At increasing doses of exosomal mimetics produced from mesenchymal stem cells loaded with paclitaxel, the viability of cancer cells was drastically reduced in vitro. Comparing paclitaxel-loaded mesenchymal stem cell-derived exosomal mimetics to control and mesenchymal stem cell-derived exosomal mimetics, the in vivo tumor development was dramatically suppressed. Consequently, drug-loaded mesenchymal stem cell-derived exosomal mimetics were demonstrated to be therapeutically effective for the treatment of breast cancer both in vitro and in vivo. These mesenchymal stem cell-derived exosomal mimetics were effectively extracted utilizing straightforward techniques. Exosomal mimetics derived from mesenchymal stem cells have the potential to be utilized as medication delivery vehicles treating breast cancer [137].

## 5. Safety and Toxicity Implications of Mesenchymal Stem Cell-Based Nanocarriers In Vitro and In Vivo

A number of therapeutic superiorities have been reported regarding the applications of mesenchymal stem cell-based nanocarriers for drug delivery applications [138]. For example, these mesenchymal stem cell-based nanocarriers exhibit a higher degree of affinity for the hypoxic microenvironment found in tumors. Both in vitro and in vivo tumor growth is inhibited by the combination of nanoparticles in mesenchymal stem cells in rodent models of cancer. The covalent conjugation of nanoparticles with mesenchymal stem cells surface can greatly enhance the delivery of drug load to tumor sites. In vitro tumor growth was inhibited by gold, silica and silicates, diamond, silver, and copper nanoparticle-based anti-angiogenic systems [130,139,140]. Glycolic acid polyconjugates, for instance, have been shown to improve the drug delivery of nanoparticles and human mesenchymal stem cells. However, along with the therapeutic efficacy and long-term stability of these mesenchymal stem cell-based nanoparticles, their biosafety should also be improved for their clinical applications [94,141]. In fact, when mesenchymal stem cells are employed as the vehicles for drug delivery paradigms along with the NPs, these exhibit lower toxicities but sometimes their inefficient accumulation in tumors can be seen because of their clearance by the reticuloendothelial organ systems. To overcome these limitations and problems, internalization or conjugation of therapeutic payload-loaded nanoparticles can be rendered more efficient by encapsulating these in mesenchymal stem cells [142,143].

Till recent, some relevant clinical reports accounted the safety of mesenchymal stem cells without tumorigenesis in patients. In the meta-analysis which comprised of 36 studies, and incorporated eight clinical trials of a randomized nature having the adequate control groups, the absence of cancers was shown post transplantation of the mesenchymal stem cells (*n* = 1012 patients) [144]. As far as safety and toxicological aspects of mesenchymal stem cell-based nanocarriers are concerned, many of their aspects continue to get resolved regarding the biosafety of these mesenchymal stem cell-based nanocarriers, such as their long-term toxicological outcomes when different types of nanoparticles are combined with mesenchymal stem cells especially with their applications in cancer patients to assure their clinical safety paradigms [145,146]. One of the key aspects is loading of the optimized nanoparticle concentrations into these mesenchymal stem cells which becomes necessary for their successful translation into clinical aspects, although more insights and better and in-depth comprehensions are always necessary which could confirm their safety and minimize their adverse effects [147,148].

**Figure 5:**
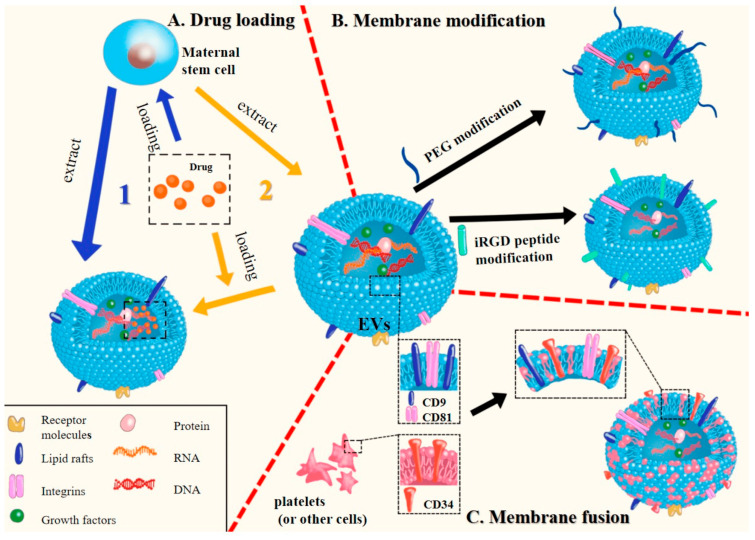
Customizations and alterations of mesenchymal stem cell-based vesicular nanostructures to establish a nano-drug delivery system: These vesicular nanostructures are obtained from mesenchymal stem cells and can be employed for formulation of drug delivery vehicles and the creation of the membranous nanocarriers appropriate for delivering the therapeutic payload (reprinted from [153]).

Mesenchymal stem cells have exhibited promising paradigms for the treatment of myocardial infarction in both animals and human studies. This regenerative medicine arena has broadly engaged engineered silica nanoparticles as the contrast agents because of their easy functionalization and resistance to degradation [149]. On the other hand, debates still remain regarding their efficacious biosafety in cell-based systems. Gallina and coworkers deeply investigated the impacts of human mesenchymal stem cells labelled with dye-loaded amorphous silica nanoparticles on the cell-viability and functional capacities and optimized the protocols of human mesenchymal stem cells labelling and also assessed their feasibility in a beating heart model. The optimized cell-labelling could be incurred after exposing these human mesenchymal stem cells to the fluorescent 50 nm nanocarriers and it was further observed that activation of lysosomes consequential to the nanoparticle reposition cannot be consociated with the oxidative stress. Long-term culturing of these human mesenchymal cells leads to preservation of their stemness/differentiation properties, proliferative capabilities, and further imparts resistance to cytotoxicity and genotoxicity. Eventually, both the ultra-structural testing of cell engraftment inside the myocardial tissues and clearer visualization of human mesenchymal stem cells in both normalized and infarcted rat hearts could be made possible by brilliant fluorescence discharged by the internalized nanocarriers. Overall, these mesenchymal stem cell-based nanocarriers exhibited superior compatibility with mesenchymal stem cells in terms of the preservation of key characteristics of these cells and the absence of cytotoxicity and genotoxicity. Because of their proven biosafety as well as their capability to label cells correctly and be seen in histological sections, these mesenchymal stem cell-based nanocarriers could provide some of the best viable routes for tracking cells within heart tissue [150]. Schematic representation of the designing and formulation strategies for safe and non-toxic stem cell-based nanostructures has been depicted in Figure 5.

Another issue is the unclear in vivo fate of these mesenchymal stem cell-based nanocarriers, which makes it challenging to establish the safety and treatment mechanism [151]. By using mesenchymal stem based inorganic nanocarriers as contrast agents, these can be made potentially feasible for their traceability, which allows for the tracking of their locations and viability in vivo. This information can be used to guide precise transplantation, clarify therapeutic mechanisms, and guarantee patient safety [152]. Another approach to enhance safety of these mesenchymal stem cell-based nanocarriers is their formulation approach viz. in the formulation of mesenchymal stem cell-based exosomes formation; direct methods which undertake passive electroporation and creation of an electric field within the membrane of the macrovesicles for improvement of their membrane permeabilities leads to enhancement of the safety of these mesenchymal stem cell-based exosomes and minimizes their potential toxicities. The other indirect methodology which involves co-incubation undertakes the modification of the parent mesenchymal stem cells with drugs followed by their transfer encapsulation inside these mesenchymal stem cells. In this way, mesenchymal stem cell-based exosomes can be formulated with special qualities—such as low immunogenicity, biosafety, nanoparticulate size, long circulation half-lives, optimal biocompatibility, exceptional penetration capabilities, and higher uptake rates—which render them perfect for biological applications in the treatment of various human diseases [148].

One key goal is to achieve clinical effectiveness of these mesenchymal stem cell-based nanomedicines with lower nanoparticle concentrations while maintaining safety and minimizing the toxicological implications. In a recent report, mesenchymal stem cell-based exosomes efficiently absorbed glucose-coated gold nanoparticles via an active, energy-dependent mechanism. The researchers tracked the labelled exosomes delivered nasally using a mouse model, and they found that within 24 h, there was a notable accumulation of these exosomes at the site of a brain injury. When compared to the control animals’ erratic movement and clearance, this accumulation was greater. The labelling technique for exosomes holds great promise as an invaluable diagnostic tool for a range of brain disorders and could enhance neuronal regeneration treatments [154]. Researchers have evaluated the safety and toxicological implications of various types of mesenchymal stem cell-based nanocarriers, the details of which have been provided in Table 2.

## 6. Biotransformation Mechanisms and Clearance of Mesenchymal Stem Cell-Based Nanocarriers

Comparing cell membrane-camouflaged drug delivery systems to cell-based drug delivery systems, recent research has revealed that the latter can lose all of the native cells’ biological characteristics and functions, including the long lifespan of erythrocytes (red blood cells, or RBCs) in circulation, the ability of macrophages, neutrophils, and mesenchymal stem cells to homing in on inflammation, and the capacity of T cells and natural killer cells to recognize and eliminate tumors [192,193,194]. Additionally to the effect of nanocarriers, various physiological and pathological barriers, such as hepatic metabolism, renal filtration, immune clearance, and various organ and tissue barriers (e.g., epithelial–endothelial barrier, extracellular matrix barrier, and cell membrane barrier), control the drug delivery capabilities of mesenchymal stem cells in vivo [195,196].

Mesenchymal stem cells as well as mesenchymal stem cell-based nanocarriers possess the larger volumes of internal spaces with hydrophilic or hydrophobic features, and due to the separate space inside the cell, they can protect the loaded pharmaceuticals from degradation and clearance for a variety of drug loadings (with distinct features) based on diverse mechanisms [108,130,197]. Drugs that are encapsulated in the cytoplasm of these stem cells can interact with other chemicals in the plasma less and be shielded from clearance and degradation before reaching their intended locations. 

Therefore, it is critical for the development and practical use of mesenchymal stem cell-based drug delivery systems to elucidate the in vivo fate of these particles, including their distribution, homing, retention, clearance, and activity [37,198,199]. Specifically, the extended half-life of the drug loaded into the bloodstream is significantly extended due to the extended half-life of these mesenchymal stem cell-based nanocarriers in circulation (60–90 days), leading to an elevated concentration of the drug in the blood. For instance, it has been observed that when the anticancer medication is administered via these nanocarriers based on mesenchymal stem cells, its circulation half-life increases from four hours to almost six days [78,200,201]. Additionally, in vivo pharmacokinetics and biodistribution of the loaded medicine in mesenchymal stem cell-based nanocarriers can be markedly enhanced by a suitably lengthy blood circulation period [202,203]. Moreover, the clearance process conducted by mononuclear phagocyte systems and other problematic tissues might occasionally result in a limitation of mesenchymal stem cell-based nanocarriers’ ability to infiltrate and passively transport medications to the liver, spleen, and other pathological sites [204,205,206].

Drug delivery methods based on mesenchymal stem cells also switch the drug’s clearance pathway from renal filtration to mononuclear phagocyte uptake. It is widely acknowledged that glomerular filtration is a common method of eliminating small-molecule medicines [207,208,209]. However, this method is not effective for compounds with a relative molecular mass larger than 70,000 or mesenchymal stem cell-based nanoparticles with a particle size bigger than 8 nm [210]. When drugs and therapeutic payloads are directly bound to mesenchymal stem cells, it can result in membrane disruption and quick in vivo clearance of drug-loaded cells. The intracellular content of mesenchymal stem cells may decrease as a result of drug loading, and the membranes and other cytological features of the mesenchymal stem cells may become less elastic, strong, and intact [211,212,213]. The circulation time of mesenchymal stem cell-based nanocarriers was negatively impacted by the drug-loading method when compared to native mesenchymal stem cells in mice and rats, but it was still significantly longer than the half-life of other nanoparticle-based drug delivery systems [214,215,216]. Furthermore, the drug-loading procedure may cause stem cells’ exposure to phosphatidylserine to rise from the normal value by several orders of magnitude (more than 5%) which leads to complement fixation and activation, dedifferentiation of mesenchymal stem cells, and elevated stress sensitivity, which ultimately impairs the biocompatibility of these mesenchymal stem cell-based nanocarriers and other drug delivery systems [217,218,219].

Mesenchymal stem cells laden with drugs can be administered locally or systemically through injections (e.g., intravenous and arterial delivery). Few mesenchymal stem cell-based nanosystems are transferred to other organs after intravenous injection; the majority of cells are first kept in the lungs and then re-distributed to the liver, spleen, and kidney [220,221,222,223]. Because of the mesenchymal stem cells’ passive adhesion in pulmonary capillaries, which is brought about by their increased cell volume and adhesion molecule expression during ex vivo culture, mesenchymal stem cell-based drug delivery systems remain in the lung after intravenous injection [224,225]. After an intravenous injection, mesenchymal stem cells can redistribute between a few minutes to several days. The half-life of mesenchymal stem cell-based nanosystems’ elimination from the lungs is roughly 10–24 h [220]. Additionally, Kraitchman and coworkers observed that in a dog model of acute myocardial infarction, mesenchymal stem cell-based systems were highly distributed in the lungs immediately following intravenous infusion and were progressively redistributed to the liver, spleen, and kidneys over the course of the next one to seven days. [226]. Consequently, effective tumor targeting may be achieved using intravenous injections of mesenchymal stem cells for the treatment of lung tumors. Mesenchymal stem cells can be surface modified to target organ and tissue delivery and extend their circulation in vivo. For instance, hyaluronic acid wheat germ agglutinin combined with mesenchymal stem cells improved targeted delivery to the liver (which expresses a lot of hyaluronic acid receptors) [227]. Varied intravenous injections can also result in varied biodistributions of mesenchymal stem cell-based nanoformulations; following portal vein injection, mesenchymal stem cell-based nanocarriers are primarily dispersed in the liver. [228,229,230]. Additionally, in comparison to the inferior vena cava injections, superior mesenteric vein injections might culminate into the in greater liver selectivity and homing times. [231].

After arterial injection, the lungs are bypassed, resulting in a greater distribution of mesenchymal stem cells in other bodily organs. When mesenchymal stem cell-based formulations were administered intravenously versus orally to pigs, the distribution of these formulations changed. After arterial injection, the amount of accumulated mesenchymal stem cells in the lungs decreased, while the amount of mesenchymal stem cell-based nanocarriers in the liver, spleen, and kidneys increased [203,232,233]. By using arterial injection, targeted distribution to certain organs and tumors can also be accomplished. Mesenchymal stem cells, for instance, injected into the kidney through the renal artery had a concentrated distribution in the kidney but not in other organs [230,234,235]. While injections into the portal vein and artery system can effectively target specific organs, these delivery modalities necessitate invasive procedures and carry a significant risk of bleeding complications. Consequently, the recommended technique for administering mesenchymal stem cells is still intravenous injection [236,237].

Different administration routes result in different in vivo fates and therapeutic outcomes for drug-loaded mesenchymal stem cell-based drug delivery systems. In addition to systemic administration, local tissue injection (e.g., central nervous systems, peritoneal, peritumor, and intratumor) is another common delivery method [238,239]. Targeted tumor locations can be effectively supplied with mesenchymal stem cells through intratumoral and peritumoral administration. Delivery via the central nervous system makes it possible to reach specific brain areas or tumors, including glioblastomas. Following intracerebroventricular injections, Wang et al. demonstrated that mesenchymal stem cells laden with paclitaxel may move and infiltrate gliomas [240,241,242]. The glioblastoma model mice’s survival was significantly extended by the cerebral injection of mesenchymal stem cells expressing interferon beta, as opposed to the intravenous treatment [243,244,245]. Furthermore, a study has reported that significant intracerebral migration can be achieved by administering mesenchymal stem cells intranasally, avoiding the blood brain barrier [246,247,248,249,250]. Various studies summarizing the various mesenchymal stem cell membrane-coated nanosystems along with their in-vivo behavior have been listed in Table 3.

## 7. Pharmacological and Immunological Barriers in Stem Cell Membrane-Based Nanocarriers

The unique qualities of stem cell membrane-based nanocarriers, such as their low immunogenicity, biocompatibility, and biodegradability, have drawn attention in the field of drug delivery. For their successful implementation, pharmacological and immunological barriers still present some difficulties that need to be overcome for their successful applications in preclinical and clinical settings.

### 7.1. Challenges in Parenteral Delivery and Biodistribution

Drug delivery has become more commonplace thanks to the development of many mesenchymal stem cell-based nanocarriers by researchers. Exosome-based nanocarriers have shown to be a great natural nanocarrier system in this series, and they can overcome the drawbacks of earlier nanocarrier-based drug delivery systems [257]. These can be released by physiological processes or pathological conditions in various types of cells including mesenchymal stem cells. The most promising exosome dimensions for sophisticated and targeted drug delivery are currently garnering increased attention due to their nanoscale nature [258]. Research has shown that exosome-based nanosystems have the best stability of any extracellular vesicle and an extraordinary ability to maintain the stability of their payload. Numerous pieces of evidence suggest that exosomal materials and nanosystems can withstand degradation caused by digestion and other biological processes, enabling them to reach their intended locations in an active state [259]. One of the key factors influencing the biodistribution and toxicity of exosomal nanosystems is their biological origin. It has been shown that tumor-derived exosomal nanosystems are capable of successfully transporting anticancer treatments to the tumor that gave rise to them. Even though tumor-derived exosomes continue to offer several benefits for tumor targeting, systemic injection of these may raise safety concerns viz. exosomes produced from tumors have the potential to stimulate tumor growth in normal tissues by initiating the establishment of pre-metastatic niches in those tissues [260,261]. According to Mirzaaghasi et al., a significant portion of exosomal nanosystems could also become transferred to the lungs in sepsis-induced mice after intravenous infusion, with over 30% moved to the lungs within one hour of injection, but almost none could be observed in the lung of healthy mice. It was found that these exosomal nanosystems could also get retained in blood stream for longer durations and could then sometimes also lead to liver failure [262].

Additionally, concerning the overcoming of the challenges of parental delivery of cell based nanocarriers, nanoparticles can improve the exosomes’ capacity to carry drugs. Lin et al. created a type of hybrid nanoparticle combining exosome and liposome by straightforward incubation, which effectively encapsulates big plasmids, in order to address the issue of the exosomes’ limited efficacy in encapsulating large nucleic acids. Moreover, nanoparticles for drug delivery have several advantages for exosomes like, they may change the biological characters of exosomes viz. and these can regulate the release of exosomes form parental cells and also regulate the contents of exosomes. Nanoparticles for drug delivery can be combined with exosomes for overcoming several of their other deficiencies including but not limited to encapsulation of larger sized therapeutic payloads [263,264].

Following parenteral administration, these nanosized delivery systems have been demonstrated to distribute to several major organs; the origin of this distribution, which is largely dependent on the chemistry of the exosomal membranes, signifies the molecular signatures required for cellular interaction as well as the subject’s pathophysiological state. According to biodistribution studies, injecting more than 400 μg of exosomal nanosystems causes the test animal to asphyxiate due to unintended aggregations and is then followed by their accumulation in the lung tissues [265]. Effective dosage reduction can be achieved by systemic administration of extracellular vesicle-based drug delivery systems; however, when accompanied by the increased tissue/organ distribution, it sometimes becomes more difficult to limit off-target binding and off-target effects [266,267]. Moreover, if the target tissue is more accessible, experimenting with different delivery methods of these mesenchymal stem cell-based nanosystems might aid in lowering the effective dose of the drug or therapeutic payload loaded in these. In fact, boosting the uptake of these mesenchymal stem cell-based nanosystems by a targeted organ can increase their efficacy, since the route of delivery will also control their biodistribution [268]. In contrast to intravenous injection, the intraperitoneal or subcutaneous route of these nanocarriers’ deliveries may further a greater accumulation in the gastrointestinal tract and pancreas, whereas the liver and spleen may sometimes exhibit lower quantities. In addition, the uptake of these mesenchymal stem cell-based nanosystems may sometimes be enhanced by the simultaneous presence of some of the extracellular proteins, e.g., albumin. Furthermore, the inverse relationship between intravenous injection of rising nanocarriers’ concentrations and their accumulation in the liver suggests that the doses of these stem cells based nanosystems can also influence their biodistribution [269,270].

Recent research has shown that there are several challenges to be solved in the parenteral dispersion of MSC-based nanocarriers. In a recent study, Gupta and colleagues [271] looked at parenteral delivery issues and emphasized the challenges of maintaining mesenchymal stem cells’ viability and functioning during the encapsulation process. The capacity of MSCs to function as therapeutic cells may be impacted by potential impacts on cell survival, which is an exciting subject raised by the encapsulation of MSCs within nanocarriers. The immune response is a significant additional issue. According to researchers, responses triggered by the immune system’s recognition of mesenchymal stem cells and their nanocarriers may compromise the therapeutic efficiency of these cells. The effectiveness of parenteral distribution is contingent upon immune response control and management strategies that ensure the survival and functioning of MSCs [272,273].

Other researchers claim that a key component has always been the biocompatibility of stem cell-based nanocarriers. Mesenchymal stem cells and stem nanocarrier materials have different compatibilities, which affects the safety and effectiveness of parenteral distribution. Minimizing undesired cytotoxic effects or interfering with the biological processes of encapsulated mesenchymal stem cells is necessary for a successful clinical translation. Achieving customized dispersion to tissues remains a challenging endeavor. The challenges of creating nanocarriers to improve mesenchymal stem cell homing and retention have been studied by researchers. To guarantee accurate targeting to the desired region while minimizing off-target consequences, new strategies are required. These strategies include surface modifications and functionalizing nanocarriers for improved tissue-specific homing in stem cells [274].

Similarly, additional challenges include biodistribution and clearance kinetics, as highlighted by some recent reports [275,276]. The nanocarriers affect the body’s ability to distribute mesenchymal stem cells, as well as the cells’ endurance and ability to go to certain locations. Understanding and improving these features is essential to enhancing the therapeutic outcomes of mesenchymal stem cell-derived nanocarrier delivery systems. In conclusion, there are a number of challenges to be solved in the parenteral administration and distribution of mesenchymal stem cell-based nanocarriers. These challenges include immune response and cell survival issues, as well as issues with the nanocarriers’ biocompatibility, accurate targeting, and biodistribution kinetics. To overcome these challenges, a complex, multidisciplinary approach including expertise from several scientific domains is required. As researchers strive to comprehend the intricacy more thoroughly, new methods ought to emerge, propelling the field’s objective of completely actualizing the therapeutic potential of mesenchymal stem cell-based nanocarriers in clinical settings.

### 7.2. MSC-Based Nanocarriers’ Stabilities in Systemic Circulation and Their Clearance

NPs have been applied to a variety of specialized conditions, including additional therapeutic uses and site-specific medication delivery systems [277,278]. They have also been utilized to prevent tumor growth. However, using mesenchymal stem cells as drug delivery vehicles with NPs can cause toxicity, ineffective accumulation in tumor locations, and potential clearance by reticuloendothelial organs. To address these issues, methods for internalizing or conjugating drug-loaded nanoparticles in mesenchymal stem cells have been proposed [49,130]. Promising outcomes have also been observed when using mesenchymal stem cells as cell-based drug delivery vectors for tumor-homing cancer treatment. But mesenchymal stem cells’ widespread biodistribution also makes non-target peripheral tissues potentially hazardous [279]. Unlike synthetic nanocarriers, mesenchymal stem cell-based nanocarriers’ can withstand engulfment or degradation while in circulation. Because they are naturally secreted chemicals, they circulate in the receiver with intrinsic stability and can cross natural barriers like the blood-brain barrier and they are less immunogenic than other conventional carriers.

Leukocyte homing to inflammatory areas is the first method for cellular trafficking via systemic circulation to be defined. This process involves a multistep adhesion and extravasation cascade. It is not unexpected that mesenchymal stem cells are believed to use comparable processes to move toward inflammatory cues arising from sites of tissue injury, including the tumor microenvironment, given their function in controlling the immune response as a whole [280,281,282]. Bypassing the first-pass effect, intra-arterial infusions can offer a single exposure to peripheral tissues and one pass through the systemic circulation before reaching the lungs. For instance, in one study, downstream micro vessels (7 μm diameter) of the cremaster muscle showed immediate stoppage at the precapillary level in >90% of mesenchymal stem cells injected into the iliac artery [49,283]. Similarly, minutes after the injection, the great majority of mesenchymal stem cells given intravenously are quickly removed from the blood and discovered in the lung’s capillary beds. This quick trapping is followed in both human and animal models by removal from the lungs and accumulation in the liver and spleen over the course of the next few hours to days. According to recent data, this “redistribution” might be the result of nonclassical phagocytic monocytes consuming cellular debris from apoptotic mesenchymal stem cells that are confined in the lungs, together with tracking markers [28,284,285]. Nevertheless, the use of mesenchymal stem cells as drug delivery vehicles with NPs may result in toxicity, ineffective accumulation in tumor areas, and potential clearance by reticuloendothelial organs. To address these issues, various methods for internalizing or conjugating drug-loaded nanoparticles in mesenchymal stem cells have been proposed [130,286].

Recent studies have shed important light on the stability of nanocarriers based on mesenchymal stem cells in systemic circulation. The authors of a study by Zhang and coworkers [287] investigated how stability was affected by surface changes on nanocarriers. They showed how appropriate surface engineering, such as covering nanocarriers with biocompatible polymers, greatly increased resistance to physiological stressors encountered in circulation and prevented early degradation, which in turn greatly improved stability. Researchers found that using sophisticated biomaterials with great structural integrity improved stability during systemic circulation. The study underlined how important it is to give careful design considerations to guarantee the structural stability of nanocarriers in physiological settings.

Together, these results highlight how crucial surface alterations and nanocarrier architecture are to maintaining the stability of mesenchymal stem cell-based nanocarriers in the intricate systemic circulation environment. As the field develops, these insights help create more robust nanocarriers that provide better stability and increase the possibility of delivering mesenchymal stem cells in therapeutic applications in a targeted and efficient manner [153,258]. Comprehensive understanding of the clearance mechanisms of nanocarriers based on mesenchymal stem cells is still a problem, despite continued research in this area. This complex procedure is clarified by several published studies. The clearance dynamics of mesenchymal stem cell-loaded nanoparticles were investigated in another study by researchers, which emphasized the function of the mononuclear phagocyte system in the quick identification and elimination of nanocarriers from circulation. Furthermore, another research emphasized how surface changes of nanocarriers affect clearance rates. Their study revealed that immune identification and subsequent clearance might be evaded by surface engineering techniques like PEGylation, which would extend the circulation duration [53,288].

Additionally, researchers have also explored the significance of nanocarrier size in clearance kinetics. According to the study, smaller nanocarriers circulated for longer periods of time, which may have helped them avoid being recognized by macrophages and lowered clearance rates. The intricacy of mesenchymal stem cells -based nanocarrier clearance is highlighted by these combined investigations, highlighting the necessity of customized design approaches to maximize circulation and improve therapeutic efficacy [37,208,279]. Understanding the subtleties of clearance pathways through these investigations helps researchers refine nanocarrier designs for enhanced stability and extended circulation in systemic settings.

### 7.3. Microenvironmental Heterogeneities and Nanoformualtion Uptake and Cellular Internalization

Mesenchymal stem cells’ behavior is significantly impacted by the physicochemical characteristics of a cellular micro as well as the nano-environment, including the effects of topography and matrix elasticities on the differentiation process. The final destinies of mesenchymal stem cells could well be determined in large parts by the chemical signals that are produced on these matrices by growth factors and other regulators [289,290]. The formulation and development of functional biomimetic scaffolds can exhibit greater promises in mesenchymal stem cell-based nanotherapeutics by offering the highly coordinated physical and chemical cues in space and time. Advancements in these sectors require an implicit comprehension of the molecular properties of the cellular-environment along with their nano interactions in order to manipulate and harness them for the development of sophisticated next-generation mesenchymal stem cell-based nanocarriers [291,292]. Researchers have documented the evolution of diverse routes and processes concerning matrix adherence and their potential correlation with stemness and stem cell differentiation. Super-resolution imaging and single molecular tools for in-vitro nano-manipulation have made it easier to identify and characterize the molecules and the mechanics of structural transformations within mesenchymal stem cells and matrices. These advancements facilitate the exploration of the mesenchymal stem cell niche and aid in the development of new classes of mesenchymal stem cell-based nanoformulations that facilitate the production of “used biomaterials” for applications in tissue engineering and regenerative medicine [293,294].

Extracellular vesicular nanostructures inferred from the mesenchymal stem cells exhibit significant promise as nanotherapeutics due to their capacity to both stimulate the immune system and promote regeneration. Phagocytic cells have been criticized for their rapid clearance of extracellular vesicular nanostructures that are foreign to them. When specific media-acquired proteins are concurrently adsorbed on the surface of extracellular vesicles, researchers have examined the effects of these proteins in the form of protein corona on these vesicular nanostructures from the mesenchymal stem cells [63,295]. These mesenchymal stem cells derived from the extracellular vesicular nanostructures are formed under two distinct culture conditions. It has been well known that the formation of the protein corona around the mesenchymal stem cell-derived nanoparticles upon systemic exposure can affect the in vivo fate of these nanoparticles. The adsorption of proteins on these mesenchymal stem cell-derived extracellular vesicular nanostructures derived from conditioned culture medium and/or after exposure to serum has not been extensively studied. It has also been reported that human monocyte-derived dendritic cells’ proinflammatory responses are mediated by the protein corona on THP-1-derived vesicular nanostructures in vitro [63,296,297].

Various kinds of other sub-populations of the bone marrow-inferred mesenchymal stem cells, which also include multipotent types of adult progenitors, marrow-obtained adult types of the multilineage showing inducible cellular structures, and very small types of the embryonic-like stem cells, may find their applicability as the drug payload delivery agents for the nanoparticles homing in these cells [298,299]. It has been quite critical to preserve the viabilities of the mesenchymal stem cells post their integration along with the drug-loaded nanoparticles and to ensure that these medications have not altered the cellular structures before they have reached their intended destination. Inducing the expression of multidrug-resistant protein 1 in these mesenchymal stem cells is one way to provide resistance to chemotherapy. There have been several administration routes for delivering these mesenchymal stem cell-based nanocarriers to their targeted destinations in vivo which include the intravenous route of administration, intraperitoneal administration, administration of the intrathecal injections, or an intravascular delivering methodology can also be employed. Regarding extensive applications of the intrathecal injections, although they have exhibited assuring outcomes in some reports, it has largely been restricted to some of the specialized microenvironments, particularly in solid tumors solid tumors [211,298].

In a different study, researchers investigated how the tumor penetration capabilities of the mesenchymal stem cell-based nanoformulations for malignant stem cell therapies can lead to the suppression of the transforming growth factor β signaling pathway. This report concentrated primarily on the existence of a secondary niche with a more hypoxic microenvironment where malignant stem cells reside [298,300]. This is due to research suggesting that hypoxic cells can exhibit the higher resistances to the traditional therapies well and may possess the higher likelihood of tumor regressions following any of the successful treatment paradigms. Mesenchymal stem cell-based nanoparticle drug delivery systems offer benefits in the treatment of cancer. The necessity for medications to extravasate from tumor arteries and disseminate deeper to be internalized by malignant stem cells is a barrier to the application of these mesenchymal stem cell drug delivery systems. This study employed mesenchymal stem cell-based nanoformulations that functioned as small interfering RNA carriers in conjunction with inhibitors of transforming growth factor-β receptors as a logical and efficient treatment against malignant stem cells [202,298,301].

## 8. Future Perspective and Conclusions

Nanomedicines based on mesenchymal stem cells offer a new avenue for research to treat a wide range of incurable illnesses. With the kind of intense attention and research that has been going on over the last five to ten years, mesenchymal stem cell-based therapy will soon be used commercially in every door. A number of important aspects still need to be further researched, including developing low-cost media, finding a practical large-scale cell harvesting technique, and examining differentiation efficiency. Furthermore, mesenchymal stem cell development on biodegradable scaffolds for in vivo transplantation may be beneficial.

Further studies on the interaction between nanomedicine and mesenchymal stem cells ought to be carried out in rat models of illness prior to being applied in human settings. This will enable us to better comprehend the regulation of stem cell function by mesenchymal stem cell-based nanocarriers. More crucially, though, we must comprehend contradictory results about the impact of particular metallic nanocarriers on mesenchymal stem cells’ interactions and differentiation. For instance, knowledge of the molecular pathways underlying pluripotent stem cells’ reprogramming and differentiation on different kinds of nanomedicines will provide specific details on future clinical uses against cancers. We also need to decrease the damage that different kinds of nanoparticles do to stem cells. Patients should not disregard the fact that some metallic nanocarriers have negative impacts on stem cell differentiation and proliferation. It is imperative to conduct further study on the toxicity of various nanoparticles to stem cells. Moreover, the effects of metallic nanocarriers on the growth of stem cells have not been thoroughly studied, and further research is needed to determine the toxicity of superparamagnetic iron oxide nanoparticles in stem cells.

To gain a better understanding of these strategies, mesenchymal stem cells should be employed to investigate the toxicity and homing of different nanoparticles and drug delivery systems. Further studies employing mesenchymal stem cells and nanoparticles in rodent cancer models verify the minimum toxicity before it is used in clinical settings. Nonetheless, evaluation of the biophysical effects and related biological activities of nanomaterials is necessary to find nanomedicines that do not affect the cells’ viabilities or membranous fluidities of mesenchymal stem cells. Lastly, research into improved migratory ability (homing) and novel biophysical characteristics of nanoparticle-mesenchymal stem cells may produce nanocarriers with improved cell trafficking. Enhancing nanocarriers with a greater internalization capability and no harmful side effects, together with the power to lower intracellular reactive oxygen species and peroxidation formation, should be the main goals of study.

From the author’s perspective, many factors, including ease of construction, stability based on thermal and biomechanical parameters, bioactivity in relation to interactions with other biochemical moieties in the body, immune response avoidance, and approach specificity with minimal side effects, are important to consider when selecting the best scaffold, nano-therapy, or other composite material. However, authors would like to suggest that here are still certain issues that require standardization. To summarize, a standard operating procedure must be created to enable the prompt storage and transfer of mesenchymal stem cells to distant institutions. This will ensure that mesenchymal stem cell-based treatments yield the best outcomes in terms of the survivability of the nano-composites created and the repeatability of the model about its efficacy in patients with the fewest negative effects. Critical aspects also include the patients’ willingness to accept such novel treatments and their output value. These techniques could serve as a catalyst, elevating the most promising mesenchymal stem cell therapies to the fore.

As far as authors’ perspective on exosomes is considered, due to their many therapeutic benefits, mesenchymal stem cell-derived exosomes have been widely used in the creation of innovative regeneration techniques for a variety of diseases. The use of exosomes in treatment reduces safety issues associated with the introduction of living cells by enabling cell-free therapy. Mesenchymal stem cell exosomes’ ability to promote regeneration in recipient cells has frequently been associated with their anti-inflammatory properties. Exosomes produced from mesenchymal stem cells possess immunomodulatory features that make them useful for treating a range of inflammatory and autoimmune diseases. By preconditioning the growth of mesenchymal stem cells—for example, by adding chemicals or cytokines, establishing hypoxic conditions, or introducing gene changes like CRISPR/Cas9—exosome activity can be readily altered. Since lot-to-lot variation in primary naïve mesenchymal stem cells can be partially addressed by using embryonic stem cells, preconditioning the stem cells, or removing exosomes from induced pluripotent stem cells, the authors would like to suggest that most of these problems and limitations with mesenchymal stem cell-based nano-therapy can be partially resolved. In conclusion, several studies’ findings suggest that exosomes produced from mesenchymal stem cells have considerable therapeutic potential for the management of a range of illnesses. The development of recommendations for safety and therapeutic effectiveness should hasten the application of exosomes generated from mesenchymal stem cells as regenerative agents in clinical settings.

Intact mesenchymal stem cells can still be just as successful in treating patients by loading other therapeutic molecules and minimizing the danger of cancer from genetic switching, even though mesenchymal stem cells with their genes switched on showed positive outcomes. In the current report, authors have presented the special abilities of mesenchymal stem cells in tumor inhibition and tropism; non-genetically modified mesenchymal stem cells provide a novel solution to this issue. It also demonstrates how effectively chemotherapeutic medications loaded into mesenchymal stem cells may be transported to cancerous areas. Moreover, mesenchymal stem cells may re-strict angiogenesis and induce apoptosis in tumor cells with the administration of a chemotherapeutic drug to the tumor sites, both of which work together to kill glioma cells.

The discussion of the potential uses of mesenchymal stem cell-based therapies supported by nanocarriers as drug delivery systems based on mesenchymal stem cells and vesicular nano-systems released by mesenchymal stem cells, as well as therapeutic agents with enhanced specificities, regenerative, and anti-inflammatory nano-therapeutic agents, have been concludes in this review. With the help of nanotechnology, mesenchymal stem cells with special abilities have been presented in preclinical and clinical settings today, surpassing the limitations of genetically produced mesenchymal stem cells. With positive results in the near future, this opens the door for new guidelines on stem cell-based nano-therapies.

## Figures and Tables

**Table 1:** Various types of nanocarriers and drug delivery systems employed for homings and associations with mesenchymal stem cell-based nanotherapeutic agents.

Type of MSC	Type of Nanoparticle	Example of Drug	Method for Preparation	Disease/Disorder/Application	Reference
Lipid Nanoparticles	Lipid nanocapsules	Ferrociphenol	Phase inversion temperature method	Glioblastomas	[96]
	Solid lipid Nanoparticle	Galantamine hydrobromide	Microemulsion method using hot homogenization	Alzheimer’s disease	[97]
	Lipid carrier nanoparticle	Ferrociphenol	Phase inversion temperature method	Brain tumor	[98]
	Nanostructured lipid carriers	Simvastatin	High shear homogenization	Diabetes	[107]
Polymer Nanoparticle	PLGA nanoparticles	Doxorubicin	Double emulsion method	Tumor	[101]
	Gelatin	Doxorubicin hydrochloride	Desolvation method	Human cervical cancer	[103]
	PLGA nanoparticles	Paclitaxel		Tumor	[102]
Inorganic Nanoparticle	Manganese Dioxide Nanoparticles	Paclitaxel	Coextrusion technique	Lung cancer	[105]
	Silica nanorattle	Doxorubicin	Modified Stober reaction	Tumor	[106]

**Table 2:** Safety and cytotoxicity testing implications of various types of nanocarriers employed in mesenchymal and other types of stem cells research in regenerative medicines (adapted from [155]).

S. No.	Types of Stem Cells	Driven from Species	Types of Nanomaterials	Safety and Toxicity Analysis	Dose/Concentrations	Conclusions	Ref.
1	Mesenchymal stem cells	Human	Cholera toxin quantum dots	Assessment of the cell viabilities, morphological evaluation, proliferative and differentiationcapacities	250 pm–16 nM	No deleterious outcomes were observed	[156]
2	Mesenchymal stem cells	Human	RGD peptide-conjugatedquantum dots	Assessment of proliferative and differentiation capacities	20–50 nM	No deleterious outcomes were observed	[157]
3	Mesenchymal stem cells	Human	Cadmium selenium zinc sulfide quantum dots	Assessment of the cellular viabilities, and immune-phenotypic profiling	0.75–3 μg/mL	No deleterious outcomes were observed	[158]
4	Mesenchymal stem cells	Human	Cadmium selenium zinc sulfide quantum dots	Evaluation of cellular viabilities, proliferative anddifferentiation capacities	1.625 μg	Impaired chondrogenic differentiation was seen	[159]
5	Mesenchymal stem cells	Human	Cadmium selenium zinc sulfide quantum dots	Assessment of cellular viabilities, proliferative as well asdifferentiation capacities	1.625 μg	Impaired chondrogenic differentiation was seen	[160]
6	Mesenchymal stem cells	Rat	Cadmium selenium zinc sulfide quantum dots	Cellular viabilities assessment, and evaluation of the differentiationcapacities	16 μg/mL	No deleterious outcomes were observed	[161]
7	Mesenchymal stem cells	Human	Carbon quantum dots	Cellular viabilities evaluation, and differentiation capacities and capacities to for the single cell spheres	50 μg/mL	No deleterious outcomes were observed	[162]
8	Adipocyte derived stem cells	Human	Graphene quantum dots	Cellular viabilities, and other metabolicactivities	0.5, 1.0, and2.0 mg/mL	No deleterious outcomes were observed	[163]
9	Mesenchymal stem cells	Rat	Graphene quantum dots	Cellular viabilities, proliferative anddifferentiation capacities	50 μg/mL	The osteogenic and adipogenicdifferentiation was enhanced	[164]
10	Mesenchymal stem cells	Human	Mesoporous silicananoparticles	Cellular adhesion capacities, immune-phenotypicprofiling	50 μg/mL	The adhesion capacity was enhanced along with the increased expression ofConnexin-43	[165]
11	Mesenchymal stem cells	Human	Spherical core-shellfluorescentsilica nanoparticles	Assessment of the cellular viabilities, and adipogenic differentiationcapacities	100 μg/mL	Impaired adipogenic differentiationwas observed	[166]
12	Mesenchymal stem cells	Human	Core-shell fluorescentsilica nanoparticles	Evaluation of the cellular viabilities, osteogenic differentiationcapacities	10 μg/mL	Enhanced osteogenic differentiation	[167]
13	Mesenchymal stem cells	Human	Mesoporous silicananoparticles	Assessment of the cellular viabilities, as well as the migrationcapacities	100 and 200 μg/mL	No deleterious outcomes were observed	[168]
14	Mesenchymal stem cells	Human	Dye-loaded amorphoussilica nanoparticles	Evaluation of the cellular viabilities, proliferative as well asdifferentiation capacities	50 μg/mL	No deleterious outcomes were observed	[150]
15	Mesenchymal stem cells	Human	Mesoporous silicananoparticles	Cellular viabilities evaluation, proliferative as well asdifferentiation capacities	20 μg/mL	No deleterious outcomes were observed	[169]
16	Mesenchymal stem cells	Human	Mesoporous silicananoparticles	Measurements of the cellular viabilities, followed by the differentiationcapacities	3–10 μg/mL	No deleterious outcomes were observed	[170]
17	Mesenchymal stem cells	Human	Mesoporous silicananoparticles	Evaluation of the cell viabilities, morphologies, Immuno-phenotypic profiles, proliferative as well as differentiationcapacities	20 μg/mL	No deleterious outcomes were observed	[171]
18	Mesenchymal stem cells	Human	Mesoporous silicananoparticles	Cellular viabilities assessment, immuno-phenotypicprofiling, proliferative as well asdifferentiation capacities	20 μg/mL	No deleterious outcomes were observed	[172]
19	Mesenchymal stem cells	Rat	Superparamagnetic iron-oxide nanoparticles	Cell viabilities assessment, and then differentiationcapacities	1, 5 μg/mL	Increment chondrogenic differentiationwas observed	[173]
20	Mesenchymal stem cells	Rat	Superparamagnetic iron-oxide nanoparticles complexed amylose cationized with spermin	Evaluation of the cell viabilities, rate of apoptosis, levels of the intracellular reactive oxygen species, measurements of the mitochondrialtransmembranepotentials, and differentiationcapacities	30 μg/mL	No deleterious outcomes were observed	[174]
21	Adipocyte-derived stem cells	Rat	Polyethylene glycol/poly vinyl pyrrolidone—Superparamagnetic iron-oxide nanoparticlesand Polyethylene glycol/polyethylene imine Superparamagnetic iron-oxide nanoparticles	Assessment of the cellular viabilities, followed by the assessment of the morphologies	12, 25, and 50 μg/mL	No deleterious outcomes were observed	[175]
22	Adipocyte derived stem cells	Rat	Superparamagnetic iron-oxide nanoparticles	Assessment of cellular viabilities, cellular morphologies, proliferative capacities	50 μg/mL	No deleterious outcomes were observed	[176]
23	Mesenchymal stem cells	Human	Superparamagnetic iron-oxide nanoparticles	Evaluations of cellular viabilities, as well as differentiationcapacities	25 μg/mL	No deleterious outcomes were observed	[177]
24	Mesenchymal stem cells	Rat	1-hydroxyethylidene-1.1-bisphosphonic acid coated Superparamagnetic iron-oxide nanoparticles	Assessment of cellular viabilities, cellular morphologies, differentiationcapacities	25 μg/mL	No deleterious outcomes were observed	[178]
25	Mesenchymal stem cells	Human	Superparamagnetic iron-oxide nanoparticles	Cellular viability evaluations, and assessment of cell morphologies, and differentiationcapacities	1, 10, and 100 μg Fe/mL	No deleterious outcomes were observed	[179]
26	Mesenchymal stem cells	Human	Superparamagnetic iron-oxide nanoparticles	Assessment of cellular viabilities, cellular morphologies, and differentiationcapacities	13–16 pg Fe/cell	Impaired chondrogenic differentiation was seen	[180]
27	Adipocyte-derived stem cells	Mouse	Penetrating peptide-bioconjugate-persistentluminescent nanoparticles	Cellular viabilities assessments, and evaluations of differentiation capacity	50 μg/mL	No deleterious outcomes were observed	[181]
28	Mesenchymal stem cells	Human	Purified polymernanoparticles	Assessments of cell viability, and proliferativecapacities	0, 5, 10, 20, 40 μg/mL	No deleterious outcomes were observed	[182]
29	Mesenchymal stem cells	Human	R8-Polymer nanoparticles	Cellular viabilities measurements, proliferative as well asdifferentiation capacities, tumorigenic index assessments, and immunophenotypicprofiling	10 μg/mL	No deleterious outcomes were observed	[183]
30	Mesenchymal stem cells	Porcine	Gadonanotubes; polymer nanoparticles	Cell viability measurements	10^14^ Gd3+ ions/cell	No deleterious outcomes were observed	[184]
31	hESC-CM	Human	Polymer nanoparticles	Cell viabilities assessments, and immunophenotypicprofiling	0, 2, 4, 8 × 10^−9^ M	No deleterious outcomes were observed	[185]
32	Mesenchymal stem cells	Human	Gold nanoparticles	Measurements of cellular viabilities, proliferative index anddifferentiation capacities	10^12^ NPs/mL	No deleterious outcomes were observed	[186]
33	Mesenchymal stem cells	Human	Silica-coated goldnanoparticles	Measurements of cellular viabilities, proliferative indices anddifferentiation capacities	0.0–0.14 nM	No deleterious outcomes were observed	[187]
34	Mesenchymal stem cells	Rat	Silica-coated goldnanoparticles	Cellular viability assessment, and proliferativecapacities	10^12^ NPs/mL	No deleterious outcomes were observed	[188]
35	Mesenchymal stem cells	Mouse	PEGylated goldnanoparticles	Assessments of cellular viabilities, migration capacities, proliferative indices, differentiation capabilities andcapacities for the colonization of the scaffolds	100 μg/mL	Increased migration capacities, increased differentiationof osteoclasts, and increased capacities for thescaffolds colonization	[189]
36	Mesenchymal stem cells	Human	2,2,6,6-tetramethylpiperidine-N-oxyl ConjugatedGold nanoparticles	Measurements of cell viabilities, proliferative indices anddifferentiation capacities	0.05–1.00 mM	Increment in chondrogenic differentiation, while decreased adipogenic differentiation	[190]
37	Adipocyte-derived stem cells	Human	N-acetyl cysteine modifiedgold nanoparticles	Assessments of cellular viabilities, as well as ALP activities	20 μM	Increased cell viabilities	[191]

**Table 3:** Summarized view of the reports implicating stem cell membrane as a coating for nanocarrier systems along with their in-vivo applications and behavior (adapted from [78]).

S. No	Inner Nanoparticle Core	Outer Coating Membrane	Active Pharmaceutical Compound	Method of Preparation	Animal Model	Outcome	Ref.
1	Polydopamine-coated gold silver nanoparticles	Mesenchymal stem cell membrane	-	- Hypotonic lysis - Sonication	Male golden hamsters injected with Propionibacterium acnes	Enhanced photothermal conversion efficiency, increased efficiency of cellular uptake, and increased anti-proliferative effects	[251]
2	Poly (lactic-co-glycolic acid) nanoparticles	Neural stem cell membranes overexpressing the CXC receptors	Glyburide	Freeze–thaw cycles and sonication and co-extrusion	Middle cerebral artery occlusion mice	For enhancing the therapeutic effect of glyburide	[252]
3	Liposomes	Mesenchymal stem cell membrane	Curcumin	Freeze–thaw cycles and sonication	Middle cerebral artery occlusion mice	For increasing the survival rate and prevention of the weight loss tendency	[253]
4	Poly (lactic-co-glycolic acid) nanoparticles	Adipose-derived stem cell membranes overexpressing the CXCR4 receptors	Vascular endothelial growth factor	Hypotonic lysis and sonication	Female C57BL/6 mice with hindlimb ischemia	Decreasing the uptake by macrophages, and to enhance the targeting of ischemic tissues	[254]
5	Mesoporous silica nanoparticles	Mesenchymal stem cell membrane	microRNA21	Sonication	Mice with myocardial infarction	Increasing the targeting of infarcted myocardium, and inhibition of the apoptosis of the cardiomyocytes	[255]
6	Iron oxide nanoparticles	Mesenchymal stem cell membrane	Kartogenin	Hypotonic lysis and sonication	Rats with osteochondral autograft transplantation	To increase the cartilage regeneration activity, and for enhancing the biosafety profiles	[256]

## Data Availability

The data presented in this study are available in this article.
